# SKIP controls flowering time via the alternative splicing of *SEF* pre-mRNA in Arabidopsis

**DOI:** 10.1186/s12915-017-0422-2

**Published:** 2017-09-11

**Authors:** Zhibo Cui, Aizi Tong, Yiqiong Huo, Zhiqiang Yan, Weiqi Yang, Xianli Yang, Xiao-Xue Wang

**Affiliations:** Rice Research Institute, Shenyang Agricultural University, Shenyang, 110866 China

**Keywords:** SKIP, Flowering time, Splicing factor, SEF, SWR1 complex

## Abstract

**Background:**

Similar to other eukaryotes, splicing is emerging as an important process affecting development and stress tolerance in plants. Ski-interacting protein (SKIP), a splicing factor, is essential for circadian clock function and abiotic stress tolerance; however, the mechanisms whereby it regulates flowering time are unknown.

**Results:**

In this study, we found that SKIP is required for the splicing of *serrated* *leaves and early flowering* (*SEF)* pre-messenger RNA (mRNA), which encodes a component of the ATP-dependent SWR1 chromatin remodeling complex (SWR1-C). Defects in the splicing of *SEF* pre-mRNA reduced H2A.Z enrichment at *FLC*, *MAF4*, and *MAF5*, suppressed the expression of these genes, and produced an early flowering phenotype in *skip-1* plants.

**Conclusions:**

Our findings indicate that SKIP regulates SWR1-C function via alternative splicing to control the floral transition in *Arabidopsis thaliana*.

**Electronic supplementary material:**

The online version of this article (doi:10.1186/s12915-017-0422-2) contains supplementary material, which is available to authorized users.

## Background

Correct timing of the vegetative to reproductive phase transition, or floral transition, is essential for seed setting in higher plants. Studies have shown that factors involved in the floral transition, including the polymerase-associated factor (PAF) and FRIGIDA (FRI) complexes, are integrated into such major pathways as the autonomous, vernalization, and photoperiod pathways [1, 2]. *FLOWERING LOCUS C* (*FLC*) encodes a MCMI, agamous, deficiens and serum response family (MADS)-box transcription factor that acts as a central repressor of the floral transition, and five homologs, *MADS AFFECTING FLOWERING* (*MAF*) *1* to *MAF5*, exist in Arabidopsis (*Arabidopsis thaliana*) [3–5]. The repression of *FLC* by factors involved in the vernalization and autonomous pathways activates the expression of *SUPPRESSOR OF CONSTANS 1* (*SOC1*) and *FLOWERING LOCUS T* (*FT*) to accelerate flowering. Vernalization, the acceleration of flowering by prolonged cold, epigenetically silences *FLC* through the activity of polycomb repressive complex 2, which deposits the repressive histone mark trimethylation of lysine 27 on histone 3 (H3K27me3) [6]. The autonomous pathway includes a series of activities that promote the epigenetic modification and RNA-mediated chromatin silencing of *FLC* [7, 8]. The antisense transcript of *FLC* affects the expression of the sense transcript, thereby influencing flowering time in Arabidopsis [9, 10].

In contrast, the PAF1 complex [11, 12], histone 2B ubiquitination [13, 14], histone 3 K4 and K36 methyltransferase complexes [15–18], the ATP-dependent SWR1 chromatin remodeling complex (SWR1-C) [19–21], and *FRI*/*FRI*-like genes (e.g., *FRL1* and *FRL2*) [22, 23] are involved in the activation of *FLC* to suppress the floral transition through chromatin modification or remodeling in Arabidopsis [2, 24]. The SWR1-C exchanges histone H2A for H2A.Z, producing variant nucleosomes. Photoperiod-independent early flowering 1 (PIE1), actin-related protein 6 (ARP6), and serrated leaves and early flowering (SEF) are components of the SWR1-C in Arabidopsis [24]. Mutations in *PIE1*, *ARP6*, and *SEF* confer early flowering phenotypes through the silencing of *FLC*, *MAF4*, and *MAF5* expression [19, 20, 25]. The SWR1-C is required for H2A.Z deposition at *FLC*, *MAF4*, and *MAF5* chromatin, which promotes transcription at these loci [21].

Precursor (pre)-messenger RNA (mRNA) splicing removes non-coding sequences (introns) from pre-mRNAs and joins the coding sequences (exons) together to generate mature mRNAs; thus, splicing plays important roles in regulating gene expression and increasing protein diversity in eukaryotes [26]. Splicing is catalyzed by the spliceosome, a large flexible RNA-protein complex consisting of five small nuclear ribonucleoprotein particles (snRNPs) and more than 180 types of proteins [27]. In eukaryotes, two distinct spliceosomes catalyze pre-mRNA splicing: the major spliceosome (consisting of the snRNPs U1, U2, U5, and U4/U6) catalyzes the splicing of U2-type introns with 5' splice site (5'ss) GT and 3' splice site (3'ss) AG sequences, while the minor spliceosome (consisting of the snRNPs U11, U12, U4atac, and U6atac) removes U12-type introns with AT-AC (5'ss-3'ss) termini [27, 28]. U12-type introns are rare, constituting less than 1% of all introns found in animals and plants [28, 29]. Alternative splicing is a process whereby the coding and non-coding fragments of a gene are rearranged in various ways by the spliceosome at different splice sites, giving rise to several mRNA transcripts from a single pre-mRNA. This process regulates transcriptome and proteome diversity, and it controls gene expression and function [30, 31]. As in other eukaryotes, splicing is emerging as an important process affecting plant development and tolerance to biotic or abiotic stress [31].

Genomic data show that about 42% of the intron-containing genes involved in development and biotic or abiotic stress responses in Arabidopsis are alternatively spliced [32, 33]. Alternative splicing of the tobacco (*Nicotiana tabacum*) *N* gene contributes to the control of plant disease resistance [34]. Regulator of C-repeat binding factor (CBF) gene expression 1, a cold-inducible DEAD (Asp-Glu-Ala-Asp) box RNA helicase, plays essential roles in pre-mRNA splicing, especially under cold stress conditions [35]. Regulator of abscisic acid (ABA) response 1/RNA-binding protein 25, a proline-tryptophan-isoleucine (PWI) motif- and RNA recognition motif (RRM)-containing protein, affects ABA signaling by regulating the splicing of *hypersensitive to ABA 1*, a protein phosphatase 2C gene [36]. Alternative splicing is also an important mechanism in circadian clock regulation. Recently, Ski-interacting protein (SKIP), spliceosomal timekeeper locus 1, protein arginine methyltransferase 5, and two *CIRCADIAN CLOCK*-*ASSOCIATED 1* (*CCA1*) transcripts (*CCA1α* and *CCA1β*) were shown to regulate the circadian clock through alternative splicing in Arabidopsis [37–42]. Root initiation defective 1, a DEAH-box RNA helicase associated with pre-mRNA splicing, plays important roles in meristem maintenance, leaf morphogenesis, and root morphogenesis [43]. In addition, evidence indicates that alternative splicing is involved in flowering time control in Arabidopsis. Alternative splicing of *FLOWERING TIME CONTROL LOCUS A* (*FCA*) pre-mRNA is involved in the control of the floral transition [44, 45]. RZ-1B and RZ-1C, nuclear-localized RNA-binding proteins, are involved in pre-mRNA splicing and flowering time control via interactions with serine/arginine-rich (SR) proteins [46]. AtSF1, the homolog of mammalian splicing factor 1 in Arabidopsis, interacts with U2 snRNP auxiliary factor 65a/b (U2AF65a/b) and acts as a splicing factor to regulate flowering time and ABA signaling [47].

SKIP, an SNW domain-containing protein, is evolutionarily conserved, with close homologs in yeast and mammals. In mammals, SKIP (also termed SNW1 and NCoA62) plays important roles as a transcriptional co-regulator and splicing factor [48, 49]. SKIP specifically regulates the alternative splicing and expression of *p21* by interacting with the 3'ss recognition factor U2AF65 and recruiting it to *p21* mRNA in vivo [49]. In yeast, the SKIP homolog Prp45 is a component of the Prp19-related complex (or nineteen complex), which functions as a splicing factor [50, 51]. Weak mutations in *prp45* cause defects in the splicing of *ACTIN* and other genes, resulting in temperature-sensitive growth, while strong *prp45* alleles are lethal [50, 52]. OsSKIPa, which was able to rescue the defects in *prp45* mutant yeast, regulates cell viability and stress tolerance in rice (*Oryza sativa*) [53]. In Arabidopsis, SKIP regulates cytokinin-associated leaf growth [54]. SKIP expression is induced by salt, mannitol, and ABA. Overexpression of SKIP confers tolerance to abiotic stress. By contrast, the down-regulation of SKIP causes reduced tolerance to abiotic stress during germination. SKIP activated the transcription of a reporter gene in yeast, suggesting that it regulates gene expression as a transcription factor [55]. SKIP is a splicing factor that interacts physically with the plant-specific SR protein SR45 to regulate circadian clock function. The *skip-1* mutation disrupts this clock, creating a lengthened clock period phenotype by altering the alternative splicing of *PSEUDO-RESPONSE REGULATOR 7* (*PRR7*) and *PRR9*, two genes in the morning loop of the oscillator [40]. Genome-wide defects in splicing have been observed in *skip-1* plants through RNA high-throughput sequencing, suggesting that SKIP is a splicing factor [40]. In addition to defects in the circadian clock, *skip-1* plants show pleiotropic phenotypes, including early flowering. However, the molecular and biochemical mechanisms whereby SKIP represses the floral transition remain obscure.

In this study, we revealed that SKIP plays an essential role in regulating flowering time of Arabidopsis. SKIP was found to regulate the splicing of *SEF* pre-mRNA and suppress flowering by activating the expression of *FLC. FLC* expression was reduced in *skip-1*, leading to an early flowering phenotype under long-day (16 h of light/8 h of darkness, LD) and short-day (8 h of light/16 h of darkness, SD) conditions. SKIP was also required for the normal splicing of *SEF* pre-mRNA (which encodes a component of the SWR1-C) through direct binding. Splicing defects in *SEF* were found to contribute to the early flowering phenotype of *skip-1*. Further, H2A.Z enrichment at *FLC* chromatin was reduced in *skip-1*. Our findings indicate a role for alternative splicing in regulating SWR1-C function to control the floral transition in Arabidopsis.

## Results

### SKIP is required for the floral transition and normal development in Arabidopsis

**Fig. 1:**
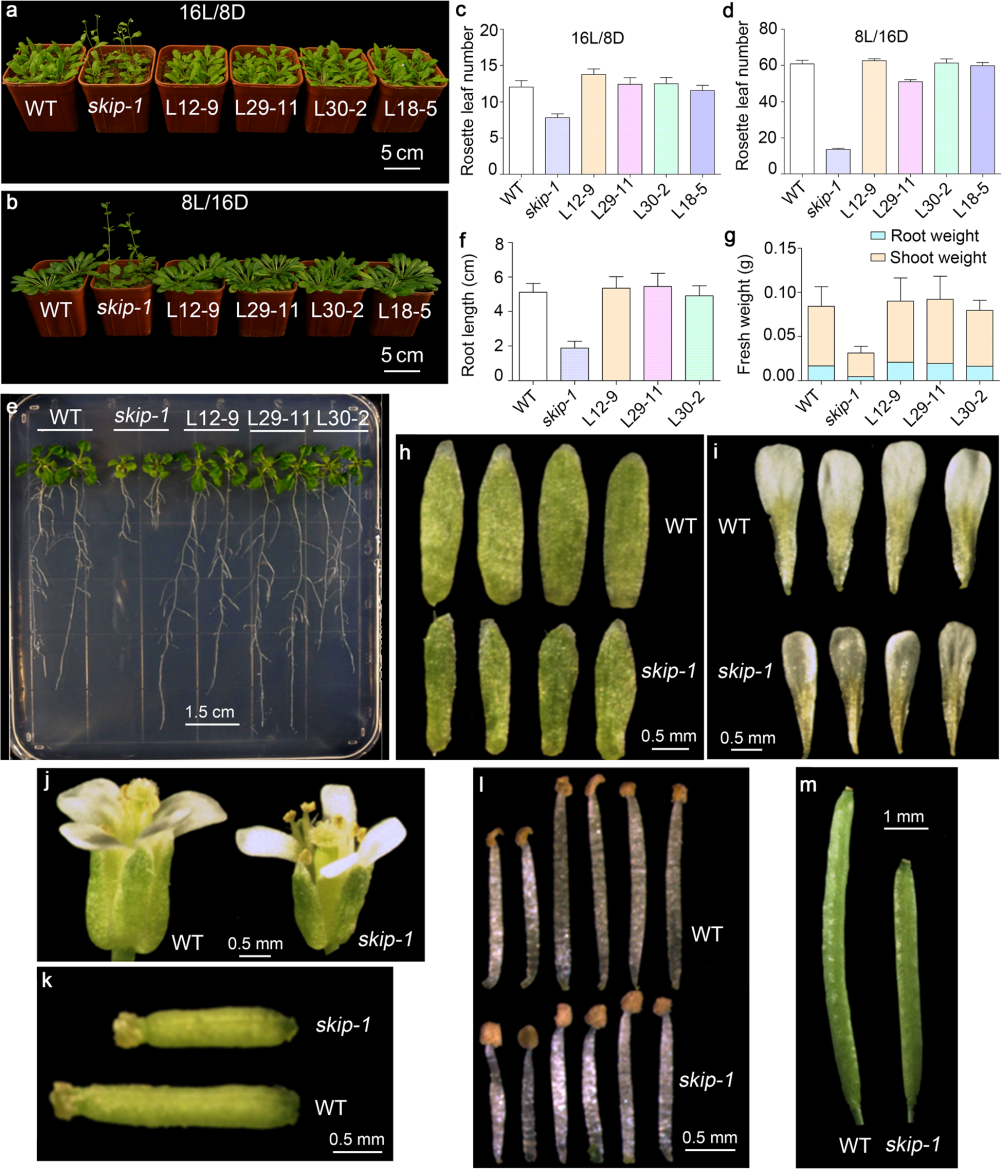
Pleiotropic phenotypes of the *skip-1* mutant. **a**–**d** Early flowering phenotype of *skip-1* under LD (**a**, **c**) and SD (**b**, **d**) conditions. **e**, **f** Inhibition of root growth in *skip-1* plants. **g** Shoot and root weights in *skip-1* mutant plants. **h**–**j** Comparison of sepal (**h**), petal (**i**), and flower (**j**) sizes in wild-type (WT) and *skip-1* plants. **k**–**m** Comparison of stigma (**k**), stamen (**l**), and silique (**m**) lengths in WT and *skip-1* plants. L12-9, L29-11, L30-2, and L18-5 are *skip-1* transgenic lines harboring the p*SKIP*:*SKIP* genomic DNA construct. All data are given as the mean ± standard deviation (s.d.) (*n* = 31–36 in **c**, *n* = 11–12 in **d**, and *n* = 34–40 in **f**). Six pools of five seedlings are represented in **g**. Scale bars are indicated in the images. 16L/8D: 16 h of light/8 h of darkness, 8L/16D: 8 h of light/16 h of darkness

Previous studies showed that the *skip-1* mutation lengthened the period of the circadian clock by impairing the alternative splicing of *PRR7* and *PRR9* in Arabidopsis [40]. The *skip-1* mutation confers an early flowering phenotype under LD and SD conditions [40]; however, the underlying molecular mechanisms are unknown. A complementation test using *SKIP* genomic DNA and *skip-1* plants revealed that the *skip-1* mutation is responsible for the observed early flowering phenotype of the mutant (Fig. 1a–d; Additional file 1: Table S1 and Additional file 2: Table S2).

Pleiotropic phenotypes were observed in *skip-1* compared to the wild type (WT), including reduced root growth (Fig. 1e and f; Additional file 3: Table S3); smaller sepals, petals, and flowers (Fig. 1h–j); shorter stigmas, stamens, and siliques (Fig. 1k–m). In the present study, we focused on dissecting the mechanisms whereby SKIP controls flowering time in Arabidopsis.

### SKIP activates the expression of *FLC* and its homologs to repress flowering

**Fig. 2:**
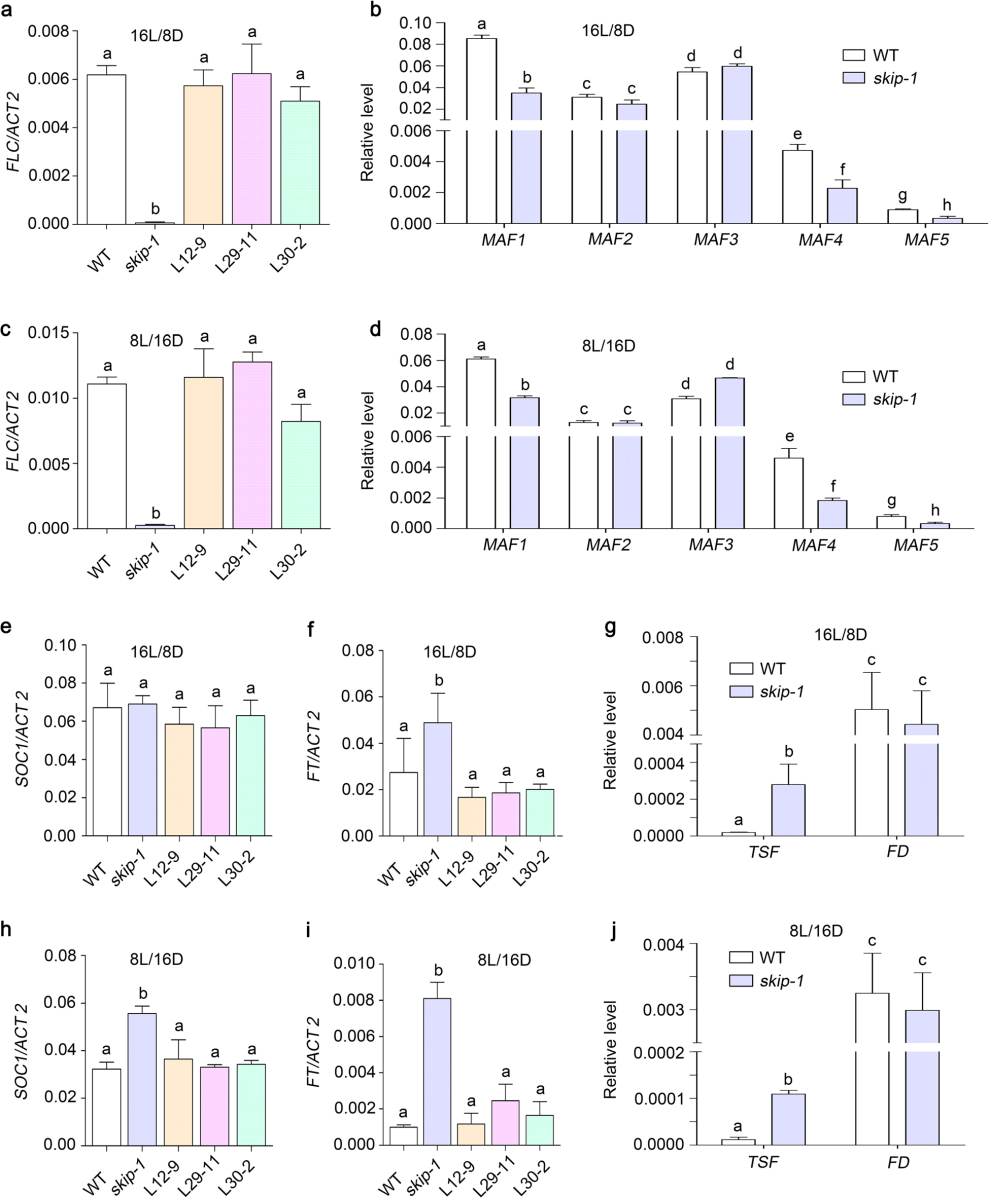
SKIP is required for the transcriptional activation of *FLC* and its homologs to repress flowering. Ten-day-old seedlings were used to examine flowering time-related gene expression by quantitative reverse transcription (qRT)-PCR. **a**, **c** *FLC* expression in WT plants, *skip-1*, and three of the *skip-1* complemented transgenic lines described in Fig. 1 under LD (**a**) and SD (**c**) conditions. **b**, **d** *MAF* expression in WT and *skip-1* plants under LD (**b**) and SD (**d**) conditions. **e**, **f**, **h**, **i** *SOC1* and *FT* expression in WT plants, *skip-1*, and the three complemented transgenic lines under LD (**e** and **f**) and SD (**h**, **i**) conditions. **g**, **j** *TSF* and *FD* expression in WT and *skip-1* plants under LD (**g**) and SD (**j**) conditions. *ACTIN 2* (*ACT2*) was used as an endogenous control. The values are the mean ± s.d. One-way analysis of variance (ANOVA; Tukey’s multiple comparison test) was performed for data in **a**, **c**, **e**, **f**, **h**, and **i**, and two-tailed unpaired *t* test was performed for data in **b**, **d**, **g**, and **j**. Statistically significant differences are indicated by different lowercase letters (*P* < 0.05). There are statistically significant differences between all non-identical letters

Because the *skip-1* mutation is insensitive to photoperiod, we examined the expression of *CONSTANS* (*CO*), a key regulator of photoperiodic flowering, in the *skip-1* mutant [56]. *CO* expression in the *skip-1* mutant was reduced or similar to that in WT Columbia (Col-0) plants under LD and SD conditions (Additional file 4: Figure S1a and b). To explore the molecular mechanisms whereby SKIP controls flowering time, we analyzed the expression of *FLC* and its homologs *MAF1* to *MAF5*, which are major suppressors of flowering, in *skip-1*. The expression of *FLC*, *MAF1*, *MAF4*, and *MAF5* was significantly suppressed by the *skip-1* mutation, leading to an early flowering phenotype under LD and SD conditions. Genomic DNA corresponding to the *SKIP* gene was able to recover the suppression of *FLC* by the *skip-1* mutation (Fig. 2a–d).

Down-regulation of *FLC* is usually accompanied by the up-regulation of downstream genes to accelerate flowering. *SOC1* and *FT* are known to accelerate the vegetative to reproductive phase transition in Arabidopsis downstream of *FLC* [2]. Twin sister of FT (TSF), an FT homolog, promotes the floral transition [57, 58]. FD, a transcription factor, interacts with FT to promote the floral transition in the shoot apical meristem [59]. Both FT and FD are required for floral meristem formation [59, 60]. As expected, the *skip-1* mutation activated the expression of *SOC1*, *FT*, and *TSF*, but not of *FD*, and it promoted flowering under LD and SD conditions (Fig. 2e–j; Additional file 4: Figure S1c). These results suggest that *skip-1* represses the expression of *FLC* and activates the expression of downstream flowering time integrators (e.g., *SOC1*, *FT*, and *TSF*) to accelerate flowering.

**Fig. 3:**
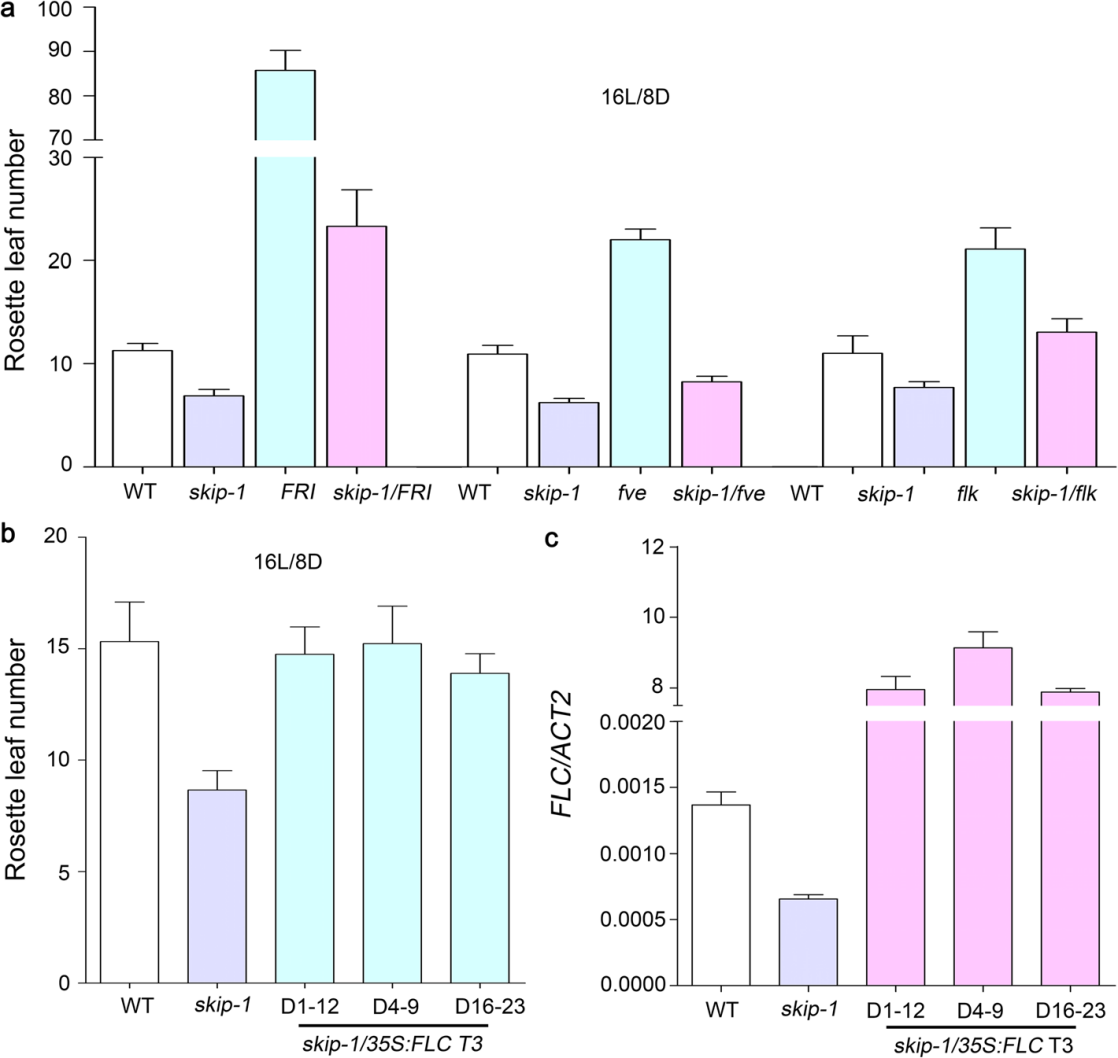
Flowering time in *skip-1*/*FRI*, *skip-1*/*fve*, and *skip-1*/*flk* double mutants, and in *skip-1* transgenic lines overexpressing *FLC*. **a** Rosette leaf number in WT, *skip-1*, *skip-1*/*FRI*, *skip-1*/*fve*, and *skip-1*/*flk* plants under LD conditions. **b** Rosette leaf number in WT plants, *skip-1*, and three *skip-1* transgenic lines (D1-12, D4-9, and D16-23) transformed with the *35S*:*FLC* construct under LD conditions. The data are the mean ± s.d. (*n* = 12–27 in **a**; *n* = 18–23 in **b**). **c** *FLC* expression as measured by qRT-PCR in WT plants, *skip-1*, and *skip-1* transgenic lines carrying the *35S*:*FLC* construct. *ACT2* was used as an endogenous control. Three technical replicates were performed. The values are the mean ± s.d.

To test whether *FLC* is genetically necessary for repression of the floral transition by SKIP, *FLC*-dependent late flowering mutants, including *FRI* (Col-0 background), *fve*, and *flowering locus k* (*flk*), were crossed with *skip-1* [22, 61–63]. The late flowering phenotypes of *FRI*, *fve*, and *flk* were dramatically suppressed by the *skip-1* mutation (Fig. 3a; Additional file 5: Table S4).

To confirm that *FLC* repression is the major mechanism that promotes flowering in *skip-1*, *skip-1* plants were transformed with a *35S*:*FLC* construct. Flowering time in the *35S*:*FLC* transgenic lines was similar to that in WT plants because of the overexpression of *FLC*, indicating that *FLC* was able to recover the early flowering phenotype of *skip-1* (Fig. 3b and c; Additional file 6: Table S5 and Additional file 7: Table S6).

Together, these genetic and molecular data demonstrate that SKIP suppresses the floral transition in Arabidopsis in an *FLC*-dependent manner.

### SKIP promotes *FLC* expression by controlling the alternative splicing of *SEF* pre-mRNA

We next investigated the mechanisms whereby SKIP regulates *FLC* expression in detail. There are three possible explanations for the silencing of *FLC* by *skip-1*. First, the *skip-1* mutation may cause the abnormal splicing of *FLC* pre-mRNA and decrease the accumulation of functional *FLC* mRNA. Second, the *skip-1* mutation may cause the overexpression of genes in the autonomous or vernalization pathway to reduce *FLC* expression. Third, the *skip-1* mutation may repress the expression of flowering time suppressors, including genes encoding members of the PAF1 complex, the SWR1-C, and so forth.

To explore the silencing mechanisms of *FLC*, *MAF1*, *MAF4*, and *MAF5* in the *skip-1* mutant, we first tested for defects in the splicing of *FLC*, *MAF1*, *MAF4*, and *MAF5* pre-mRNA in the *skip-1* mutant; except for *MAF1*, no splicing defects were detected (Additional file 8: Figure S2a; Additional file 9: Figure S6b, d, and f). Next, we assessed the mRNA expression of genes in both the autonomous and vernalization pathways, including *FCA*, *FY*, *FLK*, *FLD*, *FPA*, *FVE*, *LD*, *VRN1*, *VRN2*, and *VIN3* [24]. The expression levels of these genes in *skip-1* were similar to those in WT plants, suggesting that the *skip-1* mutation does not silence *FLC* by enhancing the expression of these genes (Additional file 8: Figure S2b and c). Finally, we analyzed the expression of floral suppressors, which were chosen according to a review [24]. Our results revealed aberrant alternative splicing and reduced levels of mature *SEF* mRNA, which encodes a component of the SWR1-C (Additional file 8: Figure S2d).

**Fig. 4:**
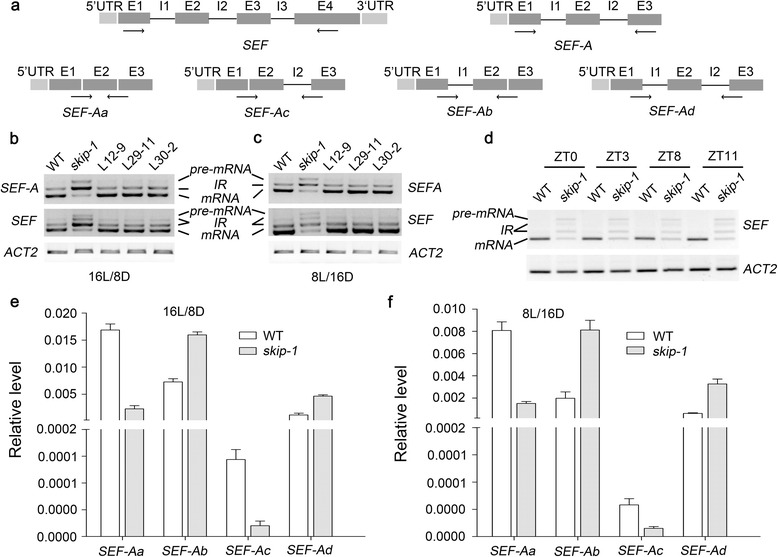
SKIP is essential for the splicing of *SEF* pre-mRNA. **a** Locations of the primer pairs used to amplify mature *SEF* mRNA and its alternatively spliced isoforms. *SEF-Aa*, *SEF-Ab*, *SEF-Ac*, *SEF-Ad* (*SEF-A*), and *SEF* represent the mature mRNA, *SEF* mRNA containing the first intron, *SEF* mRNA containing the second intron, alternatively spliced mRNA containing the first and second introns from the *SEF-A* fragment, and all isoforms of *SEF*. UTR represents untranslated region. I1, I2, and I3 represent introns 1, 2, and 3. E1, E2, E3, and E4 represent exons 1, 2, 3, and 4. The *black arrows* indicate the location of primers. **b**, **c**, **e**, **f** The levels of mature *SEF* mRNA and its alternatively spliced isoforms in WT plants, *skip-1*, and the *skip-1* complemented transgenic lines described in Fig. 1 under LD (**b**, **e**) and SD (**c**, **f**) conditions as determined by semi-RT-PCR (**b**, **c**) or qRT-PCR (**e**, **f**). **d** The levels of mature *SEF* mRNA and its alternatively spliced isoforms in WT and *skip-1* plants at different times across the circadian cycle as determined by semi-RT-PCR. ZT indicates zeitgeber time or the time under continuous light. The values in **e** and **f** are the mean ± s.d.

The expression and abnormal alternative splicing of *SEF* caused by the *skip-1* mutation were confirmed by reverse transcription-PCR (RT-PCR) and quantitative reverse transcription-PCR (qRT-PCR), respectively (Fig. 4; Additional file 10: Figure S3). Elevated accumulation of alternatively spliced *SEF* isoforms, including those showing intron retention, compared to the WT was detected in *skip-1* (Fig. 4). However, the level of mature *SEF* mRNA encoding functional SEF protein was reduced by *skip-1* (Fig. 4; Additional file 10: Figure S3). SEF has been characterized as a suppressor of flowering time and an SWR1-C component [20]. Therefore, we hypothesized that the down-regulation of *FLC* in *skip-1* mutants is caused by the abnormal splicing of *SEF* pre-mRNA.

**Fig. 5:**
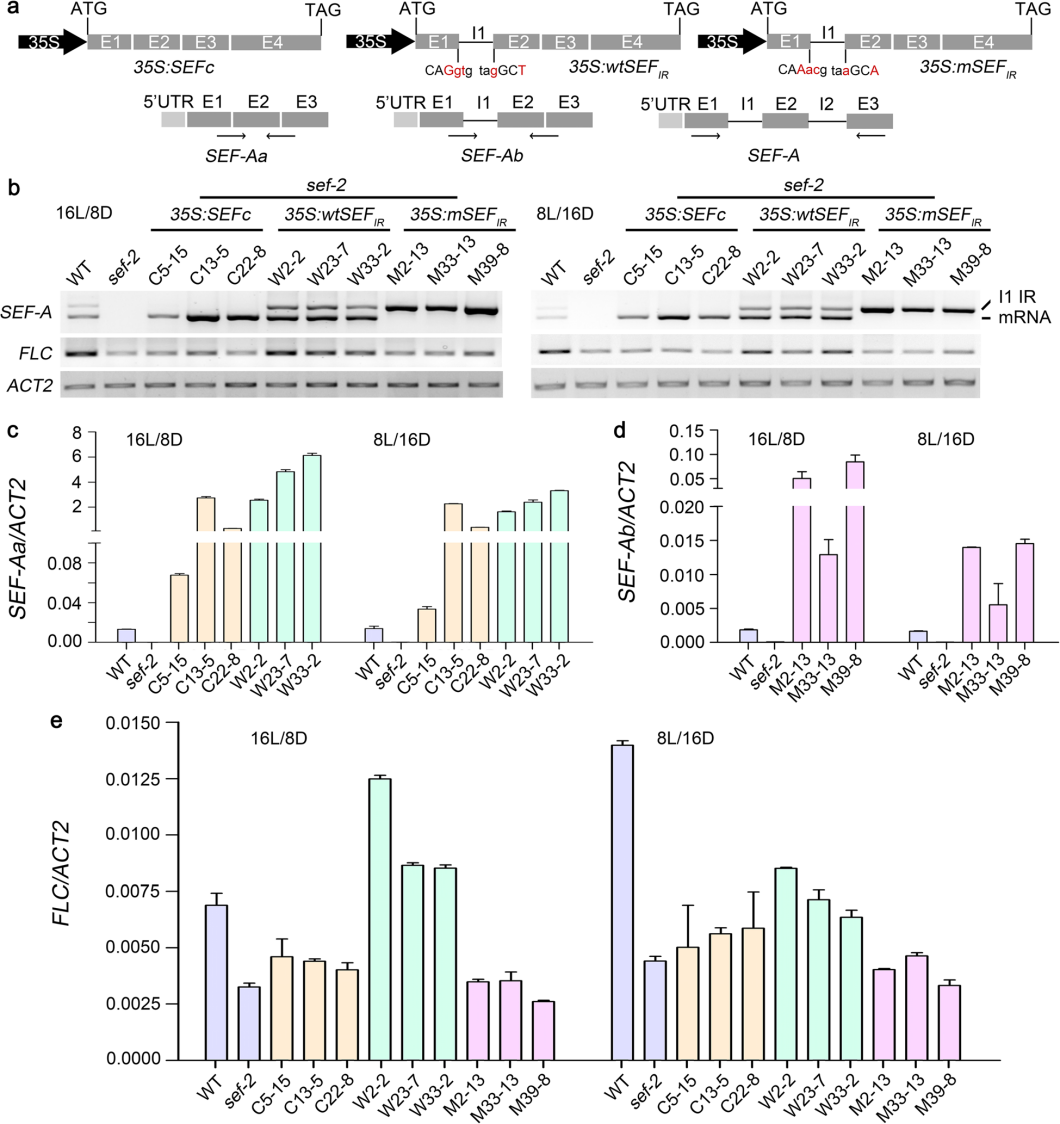
Splicing of *SEF* pre-mRNA regulates *FLC* expression. **a** Schematic representation of the constructs carrying the full-length CDS of *SEF* (*SEFc*), WT CDS with the first intron of *SEF* (*wtSEF*_*IR*_), and mutated *SEF* sequence with the first intron (*mSEF*_*IR*_) driven by the *35S* promoter. In the *mSEF*_*IR*_ construct, the 5'ss and 3'ss of the first intron were mutated, as shown in *red*. UTR represents untranslated region. I1, I2, and I3 represent introns 1, 2, and 3. E1, E2, E3, and E4 represent exons 1, 2, 3, and 4. The *black arrows* indicate the location of primers for RT-PCR. **b** *SEF* and *FLC* expression in WT plants, *sef-2*, and *sef-2* transgenic lines carrying *35S*:*SEFc*, *35S*:*wtSEF*_*IR*_, or *35S*:*mSEF*_*IR*_ under LD and SD conditions as determined by RT-PCR. **c**, **d** *SEF* expression in WT plants, *sef-2*, and *sef-2* transgenic lines carrying *35S*:*SEFc*, *35S*:*wtSEF*_*IR*_, or *35S*:*mSEF*_*IR*_ under LD and SD conditions as determined by qRT-PCR. **e** *FLC* expression in WT plants, *sef-2*, and *sef-2* transgenic lines carrying *35S*:*SEFc*, *35S*:*wtSEF*_*IR*_, or *35S*:*mSEF*_*IR*_ under LD and SD conditions as determined by qRT-PCR. *ACT2* was used as an endogenous control. The data are the mean ± s.d. (*n* = 3) in **c**–**e**. *SEF-Aa* and *SEF-Ab* represent the mature mRNA and transcript containing the first and second introns of the *SEF-A* fragment (as shown in Fig. 5a). C5-15, C13-5, and C22-8 are *sef-2* transgenic lines carrying the *35S*:*SEFc* construct; W2-2, W23-7, and W33-2 are *sef-2* transgenic lines carrying the *35S*:*wtSEF*_*IR*_ construct; and M2-13, M33-13, and M39-8 are *sef-2* transgenic lines carrying the *35S*:*mSEF*_*IR*_ construct

To test this hypothesis, we generated three *SEF* complementary DNA (cDNA) constructs: one carrying the full-length *SEF* coding sequence (CDS), the second carrying the CDS plus the first intron (*WT SEF*_*IR*_, *wtSEF*_*IR*_), and the third carrying the CDS plus a mutated version of the first intron (*mutated SEF*_*IR*_, *mSEF*_*IR*_), driven by the *35S* promoter. The *35S:wtSEF*_*IR*_ and *35S:mSEF*_*IR*_ constructs were identical except for the 5'ss and 3'ss in the first intron, which were mutated in *mSEF*_*IR*_ to produce a transcript that included the first intron (Fig. 5a). All three constructs were transformed into *sef-2* plants [20].

As expected, RT-PCR showed that, unlike *wtSEF*_*IR*_, which was efficiently spliced, *mSEF*_*IR*_ plants accumulated unspliced *SEF*_*IR*_ transcripts, which mimicked the large transcript in *skip-1* mutant plants (Fig. 5b–d).

**Fig. 6:**
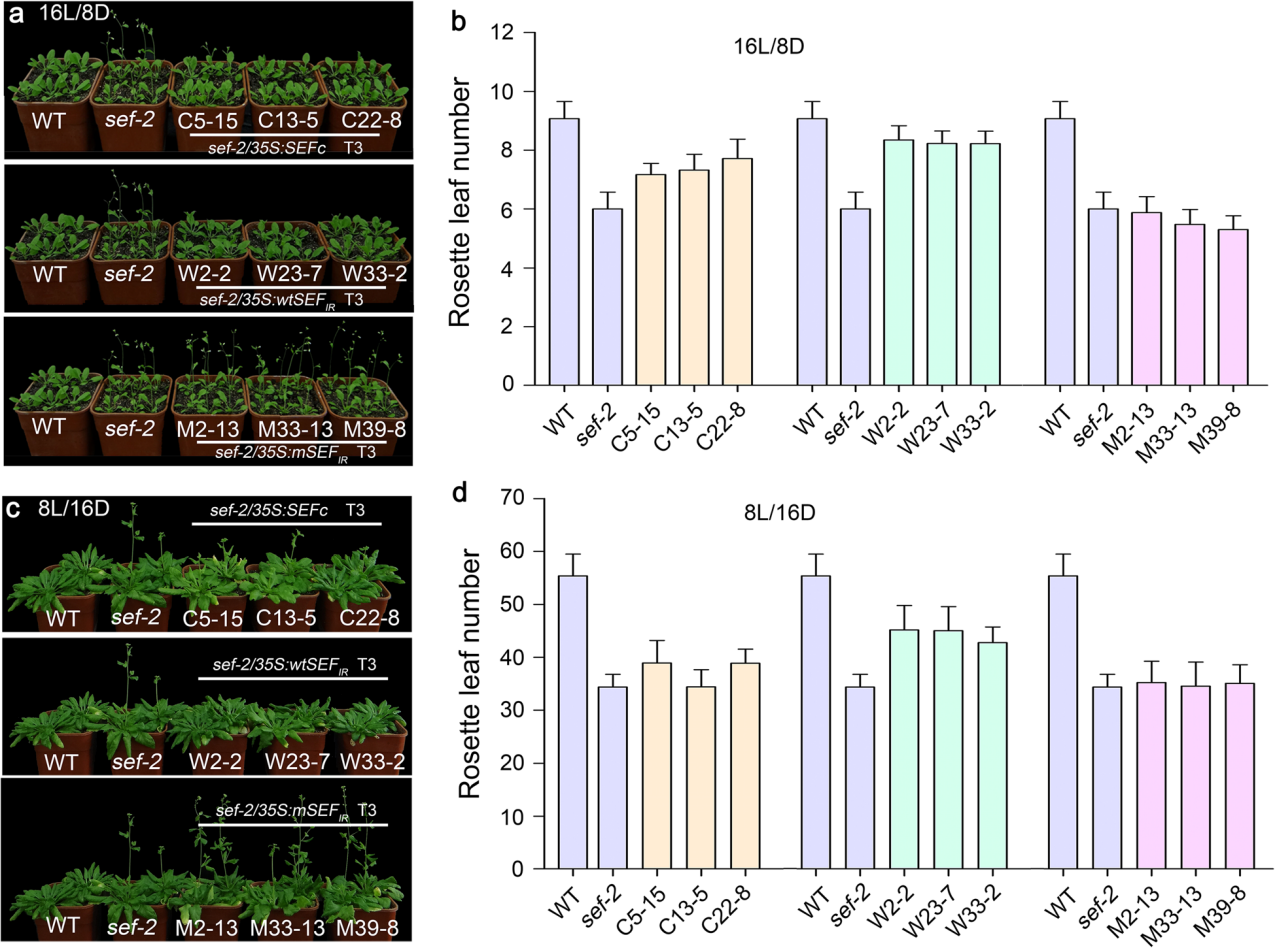
The splicing of *SEF* pre-mRNA regulates flowering time. **a**, **b** The flowering time and rosette leaf number at flowering in WT plants, *sef-2*, and *sef-2* transgenic lines harboring *35S*:*SEFc*, *35S*:*wtSEF*_*IR*_, or *35S*:*mSEF*_*IR*_ under LD conditions. **c**, **d** The flowering time and rosette leaf number at flowering in WT plants, *sef-2*, and *sef-2* transgenic lines harboring *35S*:*SEFc*, *35S*:*wtSEF*_*IR*_, or *35S*:*mSEF*_*IR*_ under SD conditions. The data are the mean ± s.d. in **b** (*n* = 20–44) and **d** (*n* = 10–16)

Transgenic plants expressing full-length *SEF* cDNA and *wtSEF*_*IR*_, but not *mSEF*_*IR*_, partially rescued the early flowering phenotype of *sef-2*, indicating that *wtSEF*_*IR*_ has a similar function to full-length *SEF* cDNA. In contrast, the unspliced form (*mSEF*_*IR*_) could not activate *FLC* expression and hence failed to delay flowering under LD and SD conditions (Fig. 5b and e; Fig. 6; Additional file 11: Table S7; Additional file 12: Table S8).

These results reveal that the derepression of *FLC* in *skip-1* was partially caused by a deficiency in the splicing of *SEF*, which is the major target through which SKIP regulates *FLC* expression (Figs. 5 and 6).

### SKIP is associated with *SEF* pre-mRNA

SKIP is localized to the nucleus [40, 55] (Additional file 13: Figure S4). During pre-mRNA splicing, the spliceosome is recruited to pre-mRNAs. Therefore, we examined whether the SKIP-containing spliceosome is associated with *SEF* pre-mRNA in vivo by RNA immunoprecipitation (RNA-IP).

**Fig. 7:**
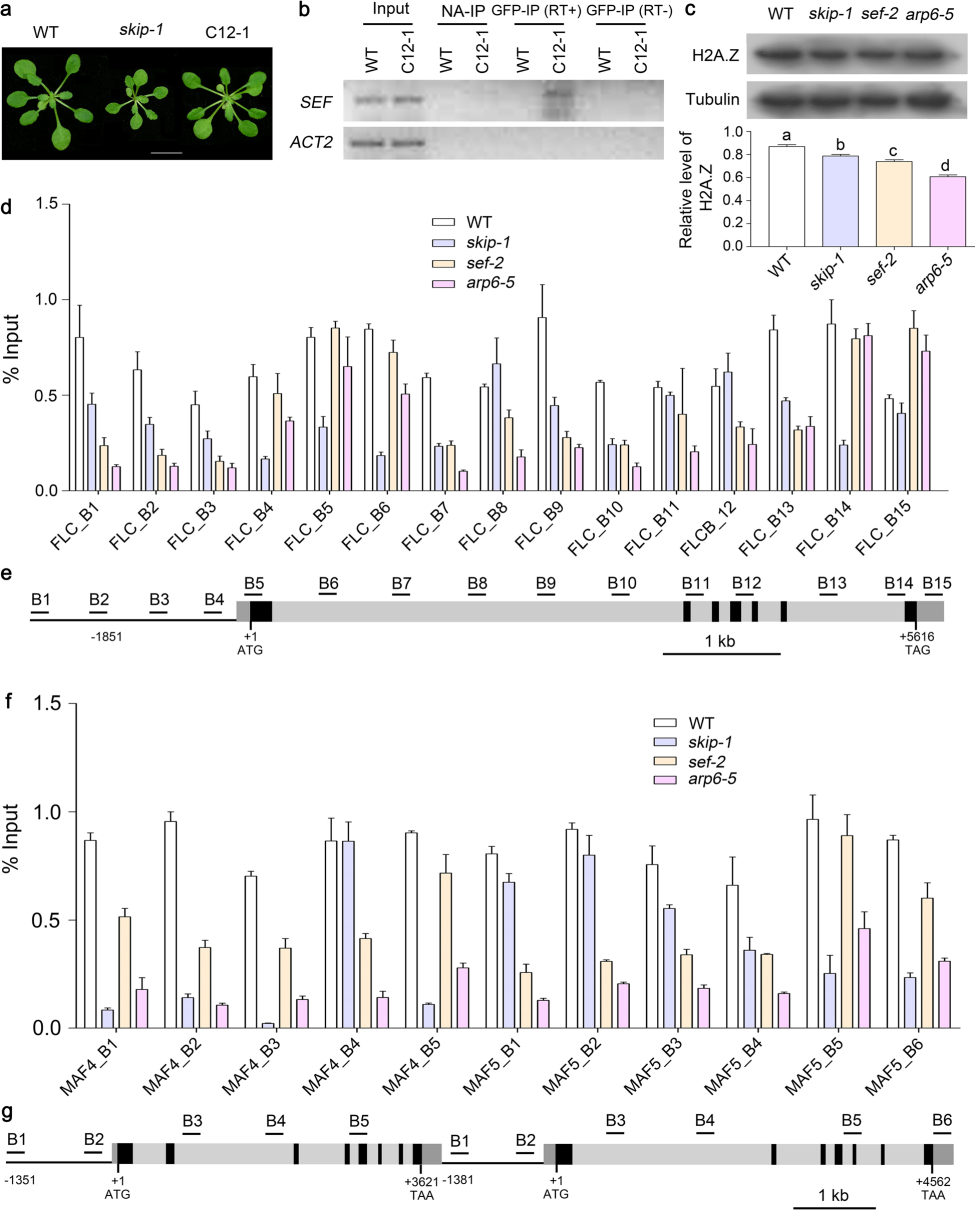
SKIP is required for the pre-mRNA splicing of *SEF* through direct binding, and it regulates the deposition of H2A.Z at *FLC*, *MAF4*, and *MAF5* chromatin. **a** Phenotypes of the plant materials used for RNA-IP. **b** RNA-IP analysis of WT and C12-1 plants. SKIP-associated *SEF* pre-mRNA was detected by RT-PCR. C12-1 is a *skip-1* complemented transgenic line harboring the *SKIP*:*GFP*-*SKIP* construct. *ACT2* was used as a negative control. Input, positive control; NA-IP, no antibody was used during immunoprecipitation as a negative control; GFP-IP (RT+) or GFP-IP (RT–), PCR was performed following RNA-IP against GFP with (RT+) or without (RT–) reverse transcription. **c** Global changes in H2A.Z deposition in WT, *skip-1*, *sef-2*, and *arp6-5* plants as detected by Western blot using anti-H2A.Z antibodies. Tubulin was used as a loading control. The relative level of H2A.Z to tubulin was quantified by Image J software. One-way analysis of variance (ANOVA; Tukey’s multiple comparison test) was performed. Statistically significant differences are indicated by different lowercase letters (*P* < 0.05). **d**, **f** H2A.Z enrichment at *FLC* (**d**), *MAF4*, and *MAF5* (**f**) chromatin in WT, *skip-1*, *sef-2*, and *arp6-5* plants as measured by ChIP with qPCR using anti-H2A.Z antibodies. The data are the mean ± s.d. (*n* = 3) in **d** and **f. e**, **g** Diagram of *FLC* (**e**), *MAF4*, and *MAF5* (**g**) with exons indicated as *black boxes*, introns indicated as *gray boxes*, and the promoter indicated as a *black line*. The PCR primer sets used are shown as *black lines* below the diagram. The names of the primer sets correspond to the numbers on the *x*-axis of the graphs in **d** and **f**. *kb* kilobases

C12-1, a transgenic line expressing a green fluorescent protein (GFP)-SKIP fusion protein (from *SKIP*:*GFP*-*SKIP*), recovered the early flowering phenotype of the *skip-1*, indicating that SKIP was functional in this line (Fig. 7a). We next used a commercial antibody against GFP to immunoprecipitate GFP-SKIP. The GFP-SKIP immunoprecipitates were then reverse-transcribed into cDNA and amplified by PCR with *SEF*-specific primers. Four sets of samples were prepared: no immunoprecipitation (Input; positive control), immunoprecipitation without anti-GFP antibodies (NA-IP; negative control), and PCR following RNA-IP against GFP with (RT+) or without (RT–) reverse transcription (Fig. 7b).

*SEF* pre-mRNA was detected in the Input and RT+ samples from line C12-1 and confirmed by sequencing (Fig. 7b). These results suggest a role for the SKIP-containing spliceosome in the splicing of *SEF* pre-mRNA.

### SKIP activates *FLC* by regulating the splicing of *SEF* pre-mRNA by replacing H2A with H2A.Z at *FLC* chromatin

SEF is a component of the SWR1-C, which is involved in the deposition of H2A.Z to target chromatin to activate gene expression [20]. To determine whether the reduced expression of *SEF* in *skip-1* plants influences the deposition of H2A.Z on a global scale, nucleoproteins were extracted from WT, *skip-1*, *sef-2*, and *arp6-5* plants and Western blot analyses were performed using anti-H2A.Z antibodies. Compared to the WT, the H2A.Z enrichment on a global scale was significantly reduced by *skip-1*, *sef-2*, and *arp6-5* mutations, indicating that SKIP may be involved in chromatin remodeling through the splicing of *SEF* in vivo (Fig. 7c).

To verify whether the reduced transcript level of *SEF* in *skip-1* affects the deposition of H2A.Z at *FLC*, we performed a chromatin immunoprecipitation (ChIP) assay to determine the level of H2A.Z across the entire *FLC* locus (Fig. 7d and e). In the WT, H2A.Z was predominantly enriched around the translation start site and first intron (regions B5, B6, and B9), stop site, and 3' untranslated region (regions B13, B14, and B15), consistent with previous data [21] (Fig. 7d). The abundance of H2A.Z across *FLC* chromatin was lower in *skip-1* than in the WT, except in regions B8, B11, and B12 (Fig. 7d and e).

To analyze whether the reduced level of *SEF* in *skip-1* was responsible for the down-regulation of *MAF4* and *MAF5*, the abundance of H2A.Z across *MAF4* and *MAF5* was examined in WT, *skip-1*, *sef-2*, and *arp6-5* plants. The H2A.Z levels across most regions of *MAF4* and *MAF5* were obviously decreased in *skip-1* (Fig. 7f and g).

These results indicate that the reduced expression of *SEF* in *skip-1* decreased the deposition of H2A.Z at *FLC*, *MAF4*, and *MAF5* in vivo to suppress their expression and promote flowering (Fig. 7d and f).

## Discussion

RNA splicing is an essential posttranscriptional process that controls gene expression and increases transcriptomic and proteomic diversity in eukaryotes. The accuracy and efficiency of pre-mRNA splicing, controlled by *cis*-acting elements and *trans*-acting factors, play important roles in regulating the expression and normal cellular function of genes [26]. The identification of splicing factors is a key step in dissecting the posttranscriptional regulation of plant development. SKIP, an SNW domain-containing protein, has been reported to be a splicing factor [40]. In this study, the mechanisms whereby SKIP controls flowering time through the alternative splicing were investigated.

### SKIP is essential for normal plant development

According to the present and previous studies, SKIP is a nuclear splicing factor that controls the circadian clock through alternative splicing in Arabidopsis [40, 55] (Additional file 13: Figure S4). Mutations in *SKIP* globally affect pre-mRNA splicing under normal growth conditions [40]. This finding is consistent with the pleiotropic phenotypes of *skip-1* observed in this study. The loss-of-function *skip-1* mutant confers early flowering phenotype under LD and SD conditions, which is consistent with the previous results [40, 64] (Fig. 1a–d, Additional files 1 and 2: Tables S1 and S2). In addition, the *skip-1* mutant exhibits multiple developmental defects, including short roots, stamens, pistils, and siliques, and small flowers, sepals, and petals, indicating that SKIP plays essential roles in plant development (Fig. 1e–m; Additional file 3: Table S3). However, the mechanisms of SKIP that regulate these biological processes remain unknown.

### SKIP is involved in the transcriptional activation of *FLC* and its homologs

The mutation in *skip-1* suppressed *FLC*, *MAF1*, *MAF4*, and *MAF5* expression, accelerated flowering by activating the expression of downstream flowering time integrators (including *SOC1*, *FT*, and *TSF*), and promoted early flowering (Fig. 2; Additional file 4: Figure S1). Consistent with this, the inactivation of *FLC* in *skip-1* partially recovered the late flowering phenotype of the *FLC*-activated mutants *FRI*, *fve*, and *flk* (Fig. 3a; Additional file 5: Table S4). Overexpression of *FLC* rescued the early flowering phenotype of *skip-1* (Fig. 3b and c; Additional file 6: Table S5 and Additional file 7: Table S6). These results provide solid evidence that the suppression of *FLC* expression is responsible for the early flowering phenotype of *skip-1*, suggesting that SKIP is required for the activation of *FLC* expression. However, the results of our pre-mRNA splicing assay and the previous report show that SKIP did not affect the splicing of *FLC* pre-mRNA, implying that SKIP indirectly regulates *FLC* expression to control flowering [64] (Additional file 8: Figure S2a).

### SKIP links the posttranscriptional regulation of SWR1-C to activation of *FLC* and its homologs

SKIP regulates the splicing of *SEF* pre-mRNA to control *FLC*, *MAF4*, and *MAF5* expression and flowering time. The *skip-1* mutation did not impact the levels of mature mRNA produced from genes encoding *FLC* suppressors and activators of the autonomous and vernalization pathways [24], except for *SEF* (Additional file 8: Figure S2b–d).

SEF is a component of the SWR1-C, which exchanges histone H2A for H2A.Z, producing variant nucleosomes. The SWR1-C is required for H2A.Z deposition at three loci: *FLC*, *MAF4*, and *MAF5*. The enrichment of H2A.Z deposition at *FLC*, *MAF4*, and *MAF5* chromatin promotes their transcription and delays flowering [21]. However, the regulatory mechanisms (especially posttranscriptional regulation) affecting the functions of the SWR1-C are unknown. In this study, we found that SKIP bound *SEF* pre-mRNA and regulated its splicing (Fig. 4 and Fig. 7b; Additional file 8: Figure S2). Our results show that the *skip-1* mutation confers defects in *SEF* pre-mRNA splicing, leading to a reduced level of mature *SEF* mRNA and elevated levels of alternatively spliced *SEF* isoforms due to intron retention (Fig. 3; Additional file 8: Figure S2d; Additional file 10: Figure S3). These isoforms of *SEF* do not function as mature *SEF* mRNAs because they cannot complement the early flowering defects in *sef-2*. Our findings show that the reduced level of mature *SEF* mRNA caused by abnormal splicing in *skip-1* inactivated the expression of *FLC*, *MAF4*, and *MAF5* and accelerated flowering. These results are consistent with studies showing that mutations in *PIE1*, *ARP6*, and *SEF* (i.e., SWR1-C component genes) conferred early flowering phenotypes in Arabidopsis by silencing *FLC*, *MAF4*, and *MAF5* expression [19, 20, 25, 65, 66]. We were unable to produce a *skip-1*/*sef-2* double mutant because of lethality, indicating that SKIP and SEF are crucial for normal plant development.

In mammals, SKIP acts as a splicing factor and transcriptional co-regulator by interacting with other proteins [48, 49]. That is the case for the ortholog of SKIP in Arabidopsis. SKIP has also been demonstrated to regulate the expression of *FLC* and *MAFs* by interacting with ELF7, a component of the PAF1 complex [64]. The expression of *FLC*, *MAF1*, and *MAF4* in *skip-1* was significantly lower than that in *sef-2*, which suggests that besides SEF, other regulators, such as ELF7, exist in *skip-1* to control the expression of *FLC*, *MAF1*, and *MAF4* (Additional file 14: Figure S5; Additional file 9: Figure S6c and e). These results reveal that SKIP plays dual roles, acting as splicing factor and transcriptional co-activator, in regulating the expression of *FLC* and *MAFs* to control flowering time in Arabidopsis.

The expression of *MAF1* in the *sef-2* mutant is similar to that in the WT, but significantly higher than that in the *skip-1* mutant; however, the splicing isoforms of *MAF1* in the *skip-1* mutant is higher than that in the WT (Additional file 9: Figure S6a and b). These results indicate that SKIP directly regulates the expression of *MAF1* through alternative splicing and the PAF1 complex [64].

### SKIP regulates the H2A.Z enrichment at the chromatin of *FLC* and its homologs to delay flowering through the splicing of *SEF* pre-mRNA

**Fig. 8:**
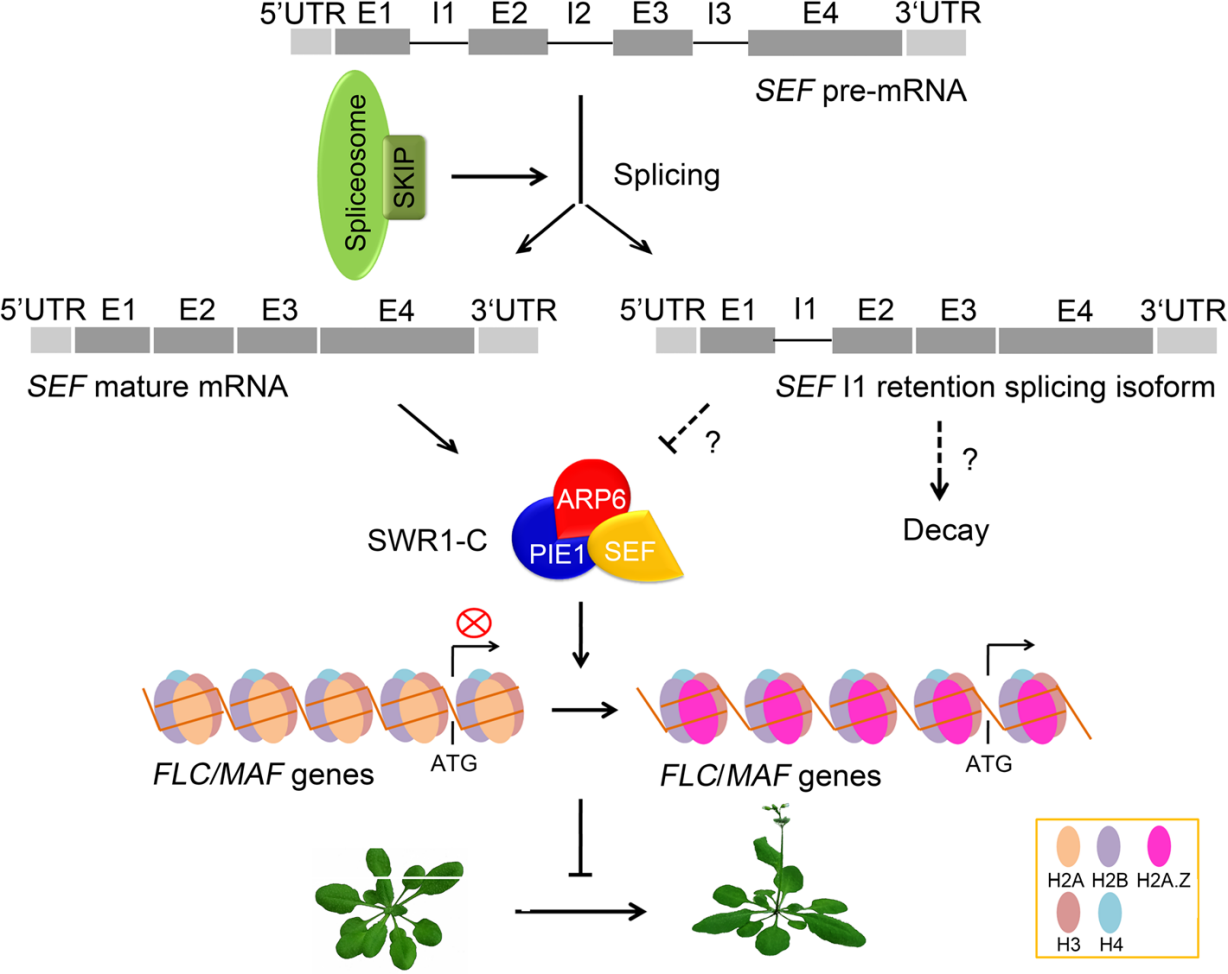
A model illustrating that SKIP is required for the pre-mRNA splicing of *SEF* to activate the expression of *FLC* and *MAF*s and delay flowering in Arabidopsis. In this model, SKIP, a spliceosome component, regulates the pre-mRNA splicing of *SEF*, which is integrated into the ATP-dependent SWR1-C. The SWR1-C regulates the substitution of H2A by H2A.Z at *FLC* and *MAF* chromatin, leading to increased *FLC*/*MAF* expression and delayed flowering in Arabidopsis. The intron 1 retention alternative splicing form of *SEF* does not function as *SEF* mature mRNA, which may be decayed through the nonsense-mediated mRNA decay (NMD) or the mechanisms unknown

Epigenetic modifications, including DNA methylation, histone methylation, acetylation, monoubiquitylation, and chromatin remodeling, of *FLC* and *MAF* have been extensively explored [2]. Previous studies showed reduced deposition of H2A.Z at *FLC*, *MAF4*, and *MAF5* in *arp6-1* and *pie1-5*, causing chromatin configuration changes and the suppression of these genes [21]. In the present study, we found that H2A.Z enrichment at *FLC*, *MAF4*, and *MAF5* was decreased in *sef-2* plants. Similarly, H2A.Z enrichment at *FLC*, *MAF4*, and *MAF5* was down-regulated by the *skip-1* mutation, resulting in the inactivation of their expression and the promotion of flowering in *skip-1* plants (Fig. 7d and f). Our findings reveal a novel mechanism whereby SKIP controls flowering time through pre-mRNA splicing and posttranscriptional regulation of the SWR1-C via ATP-dependent chromatin remodeling processes (Fig. 8). SKIP is required for the alternative splicing of the *SEF* pre-mRNA; the splicing of *SEF* pre-mRNA affects the deposition of H2A.Z at *FLC*, *MAF4*, and *MAF5* chromatin; H2A.Z deposition activates the expression of these genes to delay flowering in Arabidopsis.

It should be indicated that the levels of mature *SEF* mRNA and presumably its protein were reduced through alternative splicing but they were not knocked out in *skip-1* (e.g., in the *sef-2* mutant). It is reasonable to expect that the deposition of H2A.Z in the *skip-1* mutant would be intermediate between WT and *sef-2*; however, the level of H2A.Z at some regions of *MAF4* chromatin in *skip-1* was lower than that in *sef-2* mutant. It is possible that SEF targets substrates with differential affinities or that other unknown factors regulating the deposition of H2A.Z were inactivated in *skip-1*. This will be confirmed in future experiments.

## Conclusions

Our study reveals the regulatory roles of SKIP in flowering time control of Arabidopsis. SKIP is a splicing factor. The *skip-1* mutant exhibits an early flowering phenotype, indicating that SKIP is a suppressor of flowering time. SKIP promotes the expression of floral repressors, including *FLC*, *MAF1*, *MAF4*, and *MAF5*, and delays flowering time through regulating the pre-mRNA splicing of *SEF*. SKIP binds to the pre-mRNA of *SEF* to control its splicing and activates its expression. The elevated expression of *SEF* by SKIP accelerates the substitution of H2A by H2A.Z at *FLC*, *MAF4*, and *MAF5* chromatin, leading to increased *FLC*, *MAF4*, and *MAF5* expression and delayed flowering time in Arabidopsis.

## Methods

### Plant materials and growth conditions

All plant materials used in this study were of Arabidopsis ecotype Col-0. Seeds were sterilized and placed on Murashige and Skoog (MS) medium with 0.3% agar and 1% sucrose. After stratification in the dark at 4 °C for 2 days, the plates were transferred to white light (70 μmol m^–2^ s^–1^) in a Percival CU36L5 growth chamber (Percival Scientific, Perry, IA, USA). Plants for flowering time determination were grown under various light-dark photocycles with cool white fluorescent light (100 μmol m^–2^ s^–1^) at 22 °C during the day and 18 °C at night.

### Root elongation and fresh weight assays

Five-day-old seedlings grown in MS medium were transferred to fresh MS agar medium and allowed to grow for an additional 14 days before being harvested. The root lengths and shoot and root fresh weights were then measured.

### Complementation test

The construction of *pSKIP:SKIP* and *pSKIP:GFP-SKIP* plasmids were described in our previous report [40]. The resulting *pSKIP:SKIP* and *pSKIP:GFP-SKIP* plasmids were introduced into *skip-1* by *Agrobacterium*-mediated transformation [67]. Flowering time was examined in T3 transgenic lines (*SKIP:SKIP*/*skip-1*). Complemented T3 lines (*SKIP:GFP-SKIP/skip-1*) were used in RNA-IP.

To produce the *35S*:*FLC* construct, the 591-bp CDS of *FLC* was amplified and cloned into pCambia1300 under the control of the *35S* constitutive promoter. The resulting construct was transformed into *skip-1* plants via the *Agrobacterium*-mediated floral dip method [67]. Transformants were selected on MS medium containing hygromycin. Single insertion lines were selected based on the segregation of antibiotic resistance. Flowering time and *FLC* mRNA enrichment were examined in T3 transgenic lines.

To produce *35*:*SEFc*, *35S*:*wtSEF*_*IR*_, and *35S*:*mSEF*_*IR*_, the 516-bp CDS of *SEF* (*SEFc*), the *SEF* CDS plus the first intron (594 bp; *wtSEF*_*IR*_), and a mutated version of *SEF* with the first intron that contained an altered 5'ss and 3'ss (594 bp; *mSEF*_*IR*_) were amplified and cloned into pCambia1300 under the control of the *35S* promoter. Next, the constructs were transformed into *skip-1* via the *Agrobacterium*-mediated floral dip method [67]. Transformants were selected on MS medium containing hygromycin. Single insertion lines were selected based on the segregation of antibiotic resistance. Flowering time and the expression of *SEF* and *FLC* were examined in T3 transgenic lines.

### Gene expression assays

For RT-PCR and qRT-PCR, total RNA was extracted from 10-day-old seedlings using Takara RNAiso Plus (Takara Bio Inc., Otsu, Japan). After RNase-free DNase I (RQ1 RNase-Free DNase; Promega, Madison, WI, USA) treatment, 3 μg of RNA was used for first-strand cDNA synthesis (RevertAid First Strand cDNA Synthesis Kit; Fermentas, Waltham, MA, USA). Takara SYBR Premix Ex Taq (Takara Bio Inc.) and a 7500 Real-Time PCR Instrument (Applied Biosystems, Foster City, CA, USA) were used for qRT-PCR.

### Confocal microscopy and subcellular localization analysis

Protoplasts isolated from 3- or 4-week-old Arabidopsis leaves grown under 12 h of light/12 h of darkness as described by Asai et al. [68] and transiently transformed with *SKIP:GFP-SKIP* (constructed in pCambia1300) and roots from 7-day-old T3 transgenic seedlings harboring *SKIP:GFP-SKIP* were used for a subcellular localization analysis. Images were collected using a Zeiss LSM 510 Meta confocal laser scanning microscope (Carl Zeiss, Oberkochen, Germany) as described previously [69].

### Western blot

For immunoblotting, Arabidopsis seedlings were ground to a powder in liquid nitrogen and then homogenized in extraction buffer (10 mM Tris-HCl [pH 7.5], 150 mM NaCl, 10 mM MgCl_2_, 1% Nonidet P-40, complete protease inhibitor [Roche, Basel, Switzerland], and 1 mM phenylmethylsulfonyl fluoride). The extracts were then centrifuged, the pellet removed, and the supernatant boiled in 6X SDS sample buffer. The proteins in the samples were separated by 8% SDS-PAGE, transferred to polyvinylidene difluoride membranes (Millipore, Billerica, MA, USA), and detected using different antibodies. The antibodies used for H2A.Z and tubulin detection were anti-histone H2A.Z antibodies (Abcam Cat. no. ab4174, Bat. No. 185934-1, RRID: AB_304345; Abcam, Cambridge, UK) and anti-α-tubulin (Sigma-Aldrich Cat. No. T5168, Bat. No. 072 M4809, RRID: AB_477579; Sigma-Aldrich, St. Louis, MO, USA). The bound antibodies were visualized using Amersham ECL reagents (GE Healthcare, Little Chalfont, UK). The band intensity was analyzed with Image J software.

### RNA-IP

RNA-IP was conducted as described previously [70, 71] with slight modifications. Thirteen-day-old whole seedlings of transgenic complementation lines harboring *SKIP*:*GFP-SKIP* in a *skip-1* background and grown under LD conditions were harvested to detect the association of SKIP with *SEF* pre-mRNA. After crosslinking in 1% formaldehyde, pre-immunoprecipitation treatment, and immunoprecipitation with anti-GFP antibodies (Abcam Cat. No. ab290, Bat. No. GR197631-1, RRID: AB_303395), the immunoprecipitation products were eluted with elution buffer. The associated RNAs were quantified by RT-PCR with primer pairs crossing the intron-exon junctions in *SEF* pre-mRNA after reversal of the crosslinks.

### ChIP

ChIP was performed as described by Gendrel et al. [72] using 13-day-old seedlings grown on MS medium under LD conditions. Anti-H2A.Z antibodies were purchased from Abcam (Abcam Cat. No. ab4174, Bat. No. 185934-1, RRID: AB_304345). The primers used for qPCR are provided in Additional file 15: Table S9.

### Accession numbers

Sequence data from this article can be found in the Arabidopsis Genome Initiative database under the following accession numbers: *SKIP* (At1g77180), *FRI* (Col-0) (16-bp deletion), *fve* (SALK_013789.44.25.x), and *flk* (SALK_001523.29.35.x). These mutants were ordered from the Arabidopsis Biological Resource Center. The *sef-2* (CS841940) and *arp5-6* (CS872355) mutants were kindly provided by Dr. Ilha Lee in the Laboratory of Plant Developmental Genetics and School of Biological Sciences at Seoul National University in Korea.

## Additional files

Additional file 1: Table S1. Early flowering phenotypes of *skip-1* under LD conditions. (DOC 49 kb)

Additional file 2: Table S2. Early flowering phenotypes of *skip-1* under SD conditions. (DOC 37 kb)

Additional file 3: Table S3. Root length and fresh weight of *skip-1* under LD conditions. (DOC 34 kb)

Additional file 4: Figure S1. SKIP is essential for *FT* expression, but it is not required for *CO* expression. Ten- or 17-day-old seedlings were used for qRT-PCR analysis. a Expression of *CO* in WT and *skip-1* plants under LD and SD conditions. b Diurnal expression of *CO* in WT and *skip-1* plants under SD conditions. c Diurnal expression of *FT* in WT and *skip-1* plants under SD conditions. *ACT2* was used as an endogenous control. Three biological replicates were performed with similar results, and the result from one of the experiments is shown. The values are the mean ± s.d. (TIF 996 kb)

Additional file 5: Table S4. *skip-1* is able to recover the late flowering phenotypes of *FRI* and *fve*, and *flk* mutants under LD conditions. (DOC 43 kb)

Additional file 6: Table S5. Overexpression of *FLC* is able to recover the early flowering phenotypes of *skip-1* under LD conditions. (DOC 34 kb)

Additional file 7: Table S6. *FLC* expression as measured by qRT-PCR in the *skip-1* transgenic lines under LD conditions. (DOC 32 kb)

Additional file 8: Figure S2. Identification of factors through which SKIP activates *FLC* expression. a Measurement of *FLC* splicing defects in WT plants, *skip-1*, and the three *skip-1* complemented transgenic lines described in Fig. 1. The locations of the primer pairs used to amplify mature *FLC* mRNA and its alternatively spliced isoforms (*upper panel*). Scale bar, 1 kb. *FLC* was divided into two fragments to amplify its alternatively spliced isoforms by semi-RT-PCR under LD and SD conditions (*lower panel*). b and c Expression of genes encoding *FLC* transcriptional activators in WT and *skip-1* plants as detected by semi-RT-PCR and qRT-PCR. Three biological replicates were performed with similar results, and the result from one of the experiments is shown. The values are the mean ± s.d. in c. d The expression of genes encoding *FLC* transcriptional repressors in WT and *skip-1* plants as determined by semi-RT-PCR. *ACT2* was used as an endogenous control. IR, alternatively spliced isoforms produced by intron retention. (TIF 7388 kb)

Additional file 9: Figure S6. The expression and alternative splicing of *MAF1*, *MAF4*, and *MAF5* in *skip-1* and *sef-2* mutants under LD conditions. a, c, e *MAF1*, *MAF4*, and *MAF5* expression in WT, *skip-1*, *sef-2*, and three of the skip-1 complemented transgenic lines, including L12-9, L29-11, and L30-2, described in Fig. 1 under LD conditions. b, d, f Alternative splicing of *MAF1*, *MAF4*, and *MAF5* in WT, *skip-1*, *sef-2*, and three of the skip-1 complemented transgenic lines, including L12-9, L29-11, and L30-2, described in Fig. 1 under LD conditions. *ACTIN 2* (*ACT2*) was used as an endogenous control. Three technical replicates were performed. The values are the mean ± s.d. One-way analysis of variance (ANOVA; Tukey’s multiple comparison test) was performed for data in **a**, **c**, and **e**. Statistically significant differences are indicated by different lowercase letters (*P* < 0.05). There are statistically significant differences between all non-identical letters. (TIF 8262 kb)

Additional file 10: Figure S3. SKIP, a splicing factor, is essential for the splicing of *SEF* pre-mRNA. a Locations of the primer pairs used to amplify mature *SEF* mRNA and its alternatively spliced isoforms. *SEF-Ba*, *SEF-Bb*, *SEF-Bc*, and *SEF-Bd* represent the mature mRNA, isoform containing the second intron, isoform containing the third intron, and isoform containing the second and third introns of the *SEF-B* fragment. The *black arrows* indicate the location of primers. b and c The levels of mature *SEF* mRNA and alternatively spliced isoforms in WT and *skip-1* plants under LD and SD conditions as determined by qRT-PCR. Three technical replicates were performed. The values are the mean ± s.d. in b and c. (TIF 1341 kb)

Additional file 11: Table S7. SEF regulates flowering time through alternative splicing under LD conditions. (DOC 38 kb)

Additional file 12: Table S8. SEF regulates flowering time through alternative splicing under SD conditions. (DOC 40 kb)

Additional file 13: Figure S4. SKIP is localized to the nucleus to perform its function. a A SKIP construct encoding GFP driven by the native *SKIP* promoter (*SKIP*:*GFP*-*SKIP*) was transiently expressed in Arabidopsis protoplasts. b Roots of 7-day-old seedlings stably transformed with *SKIP*:*GFP*-*SKIP*. The images were produced by laser scanning confocal microscopy. *DAPI* 4',6-diamidino-2-phenylindole. Scale bar, 10 μm in a and 20 μm in b. (TIF 4964 kb)

Additional file 14: Figure S5. The expression of *FLC* in WT, *skip-1*, *sef-2*, and three of the *skip-1* complemented transgenic lines, including L12-9, L29-11, and L30-2, described in Fig. 1 under LD conditions. *ACTIN 2* (*ACT2*) was used as an endogenous control. Three technical replicates were performed. The values are the mean ± s.d. One-way analysis of variance (ANOVA; Tukey’s multiple comparison test) was performed. Statistically significant differences are indicated by different lowercase letters (*P* < 0.05). There are statistically significant differences between all non-identical letters. (TIF 503 kb)

Additional file 15: Table S9. Primers used in ChIP assays. (DOC 43 kb)

## Abbreviations

ARP6: Actin-related protein 6
CCA1: Circadian clock-associated 1
CO: CONSTANS
FCA: Flowering time control locus A
FLC: Flowering locus C
FLD: Flowering locus D
FLK: Flowering locus K
FRI: FRIGIDA
FT: Flowering locus T
FTIP1: FT-interacting protein 1
GFP: Green fluorescent protein
LD: Long day
MAF: MADS affecting flowering
PAF1: Polymerase-associated factor 1
PIE1: Photoperiod-independent early flowering 1
PRR7: PSEUDO-RESPONSE REGULATOR 7
SD: Short day
SEF: Serrated leaves and early flowering
SKIP: Ski-interacting protein
snRNP: Small nuclear ribonucleoprotein particle
SOC1: Suppressor of overexpression of constans 1
SR: Serine/arginine-rich
SWR1-C: SWR1 chromatin remodeling complex
TSF: Twin sister of FT
VIN3: Vernalization insensitive 3
VRN1: Vernalization 1
