# Correction to: SKIP controls flowering time via the alternative splicing of *SEF* pre-mRNA in Arabidopsis

**DOI:** 10.1186/s12915-019-0640-x

**Published:** 2019-03-20

**Authors:** Zhibo Cui, Aizi Tong, Yiqiong Huo, Zhiqiang Yan, Wenqi Yang, Xianli Yang, Xiao-Xue Wang

**Affiliations:** Rice Research Institute, Shenyang Agricultural University, Shenyang, 110866 China

**Correction to: BMC Biol (2017), 15:80**

**https://doi.org/10.1186/s12915-017-0422-2**

Upon publication of the original article [1], the authors noticed that they omitted Additional file 16: Table S10 from the Additional file list. Additional file 16: Table S10 can be found attached to this Correction and the caption of this Additional file can be found below.

## Additional file

Additional file 16: **Table S10.** Primers used in the studies (DOCX 18 kb)
