# Supplementary material for: SKIP controls flowering time via the alternative splicing of SEF pre-mRNA in Arabidopsis
Source: BMC Biol. 2017 Sep 11;15:80. doi: 10.1186/s12915-017-0422-2 (PMC5594616; doi:10.1186/s12915-017-0422-2)
Supplement: Supplementary file 1 — Early flowering phenotypes of skip-1 under LD conditions. (DOC 49 kb) [file 12915_2017_422_MOESM1_ESM.doc]

**Additional file 1: Table S1.** Early flowering phenotypes of *skip-1* under LD^1^ conditions

| Genotype | Rosette leaf number | Cauline leaf number | Day to flower bud emerging (day) | Day to first flower blooming (day) | n |
| --- | --- | --- | --- | --- | --- |
| WT^3^ | 12.06 ± 0.87^2^ | 2.83 ± 0.71 | 23.33 ± 1.46 | 30.22 ± 1.52 | 34 |
| *skip-1* | 7.82 ± 0.53 | 3.59 ± 0.62 | 19.71 ± 0.77 | 28.94 ± 0. 97 | 35 |
| L12-9^4^ | 13.76 ± 0.74 | 3.76 ± 0.55 | 27.56 ± 1.11 | 34.88 ± 1.25 | 34 |
| L29-11 | 12.42 ± 0.91 | 3.42 ± 0.73 | 23.19 ± 1.55 | 30.58 ± 2.23 | 36 |
| L30-2 | 12.52 ± 0.85 | 3.23 ± 0.43 | 24.26 ± 1.37 | 31.48 ± 1.31 | 31 |
| L18-5 | 11.56 ± 0.72 | 2.78 ± 0.49 | 21.94 ± 1.13 | 28.59 ± 1.36 | 32 |

1. LD: long day (16 h light/8 h dark); 2. The data are mean ± s.d.. 3. WT: wild type. 4. L12-9, L29-11, L30-2, and L18-5 are the *skip-1* transgenic lines harboring *pSKIP*:*SKIP* genomic DNA construct.
