# Supplementary material for: SKIP controls flowering time via the alternative splicing of SEF pre-mRNA in Arabidopsis
Source: BMC Biol. 2017 Sep 11;15:80. doi: 10.1186/s12915-017-0422-2 (PMC5594616; doi:10.1186/s12915-017-0422-2)
Supplement: Supplementary file 2 — Early flowering phenotypes of skip-1 under SD conditions. (DOC 37 kb) [file 12915_2017_422_MOESM2_ESM.doc]

**Additional file 2: Table S2.** Early flowering phenotypes of *skip-1* under SD^1^ conditions

| Genotype | Rosette leaf number | Cauline leaf number | Day to flower bud emerging (day) | Day to first flower blooming (day) | n |
| --- | --- | --- | --- | --- | --- |
| WT^3^ | 60.82 ± 1.99^2^ | 9.18 ± 0.87 | 71.82 ± 2.27 | 79.82 ± 2.56 | 11 |
| *skip-1* | 13.58 ± 0.67 | 7.00 ± 1.21 | 39.33 ± 0.98 | 56.92 ± 0.79 | 12 |
| L12-9^4^ | 62.58 ± 1.08 | 9.33 ± 0.65 | 75.00 ± 2.00 | 83.92 ± 2.64 | 12 |
| L29-11 | 51.08 ± 1.16 | 9.00 ± 0.74 | 66.58 ± 1.83 | 75.42 ± 1.68 | 12 |
| L30-2 | 61.27 ± 2.28 | 9.45 ± 0.82 | 73.00 ± 2.45 | 80.36 ± 2.29 | 11 |
| L18-5 | 59.82 ± 1.78 | 8.36 ± 0.50 | 72.00 ± 2.45 | 81.82± 1.99 | 11 |

1. SD: short day (8 h light/16 h dark); 2. The data are mean ± s.d.. 3. WT: wild type. 4. L12-9, L29-11, L30-2, and L18-5 are the *skip-1* transgenic lines harboring *pSKIP*:*SKIP* genomic DNA construct.
