# Supplementary material for: SKIP controls flowering time via the alternative splicing of SEF pre-mRNA in Arabidopsis
Source: BMC Biol. 2017 Sep 11;15:80. doi: 10.1186/s12915-017-0422-2 (PMC5594616; doi:10.1186/s12915-017-0422-2)
Supplement: Supplementary file 3 — Root length and fresh weight of skip-1 under LD conditions. (DOC 34 kb) [file 12915_2017_422_MOESM3_ESM.doc]

**Additional file 3: Table S3.** Root length and fresh weight of *skip-1* under LD conditions

| Genotype | Root length  (cm) | Root fresh weight (g) ^1^ | Shoot fresh weight (g) | Total fresh weight (g) |
| --- | --- | --- | --- | --- |
| WT^3^ | 5.12 ± 0.51^2^ | 0.0164 ± 0.0159 | 0.0677 ± 0.0438 | 0.0842 ± 0.0223 |
| *skip-1* | 1.88 ± 0.39 | 0.0046 ± 0.0042 | 0.0264 ± 0.0103 | 0.0310 ± 0.0076 |
| L12-9^4^ | 5.35 ± 0.67 | 0.0205 ± 0.0190 | 0.0694 ± 0.0445 | 0.0899 ± 0.0265 |
| L29-11 | 5.45 ± 0.76 | 0.0192 ± 0.0189 | 0.0728 ± 0.0457 | 0.0920 ± 0.0263 |
| L30-2 | 4.91 ± 0.58 | 0.0161 ± 0.0135 | 0.0633 ± 0.0333 | 0.0794 ± 0.0116 |

1. Weight of 5 seedlings; 2. The data are mean ± s.d. (6 pools of 5 seedlings). 3. WT: wild type. 4. L12-9, L29-11, and L30-2 are the *skip-1* transgenic lines harboring *pSKIP*:*SKIP* genomic DNA construct.
