# Supplementary figures and images for: SKIP controls flowering time via the alternative splicing of SEF pre-mRNA in Arabidopsis
Source: BMC Biol. 2017 Sep 11;15:80. doi: 10.1186/s12915-017-0422-2 (PMC5594616; doi:10.1186/s12915-017-0422-2)

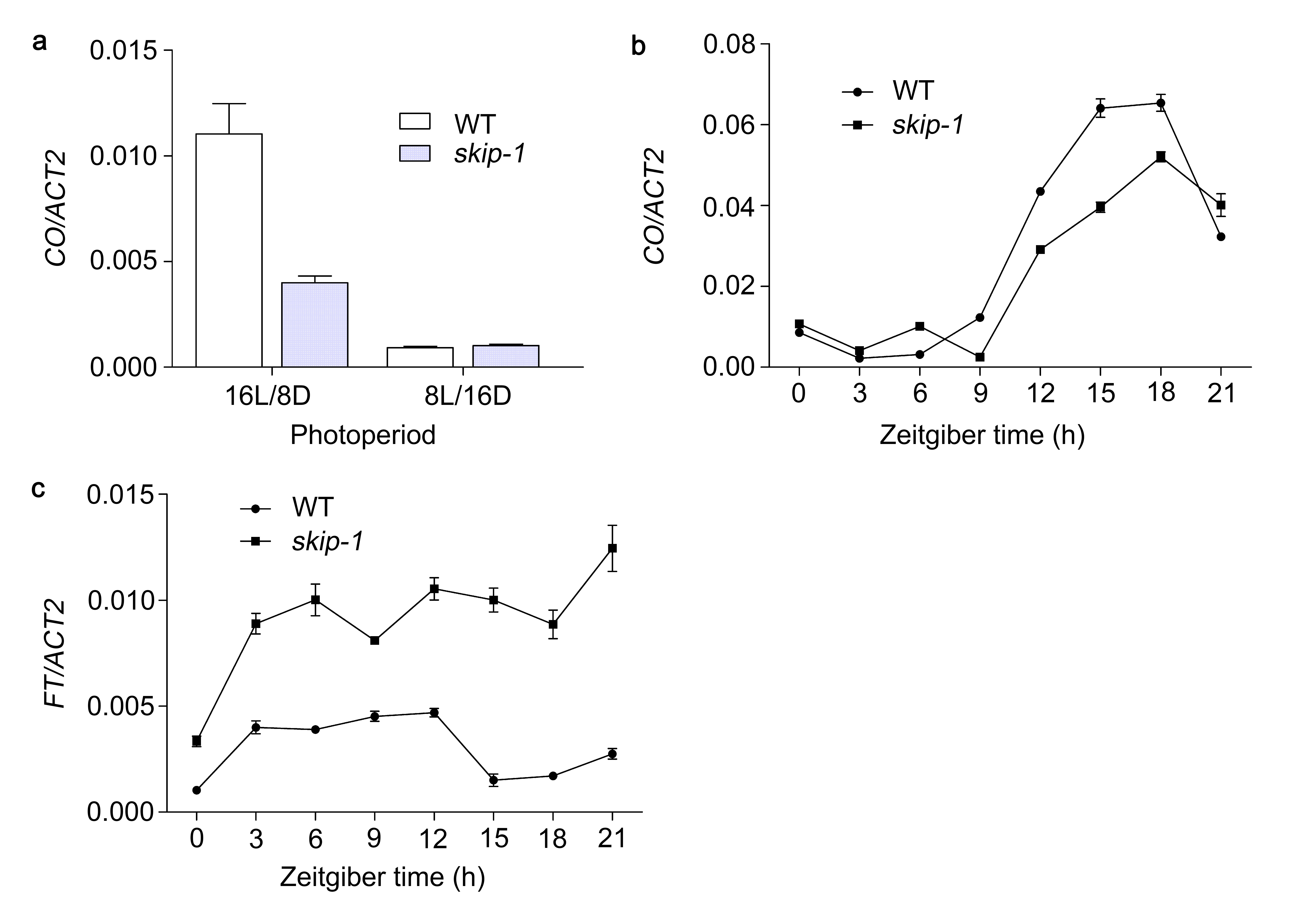

Supplement: Supplementary file 4 — SKIP is essential for FT expression, but it is not required for CO expression. Ten- or 17-day-old seedlings were used for qRT-PCR analysis. a Expression of CO in WT and skip-1 plants under LD and SD conditions. b Diurnal expression of CO in WT and skip-1 plants under SD conditions. c Diurnal expression of FT in WT and skip-1 plants under SD conditions. ACT2 was used as an endogenous control. Three biological replicates were performed with similar results, and the result from one of the experiments is shown. The values are the mean ± s.d. (TIF 996 kb) [file 12915_2017_422_MOESM4_ESM.tif]

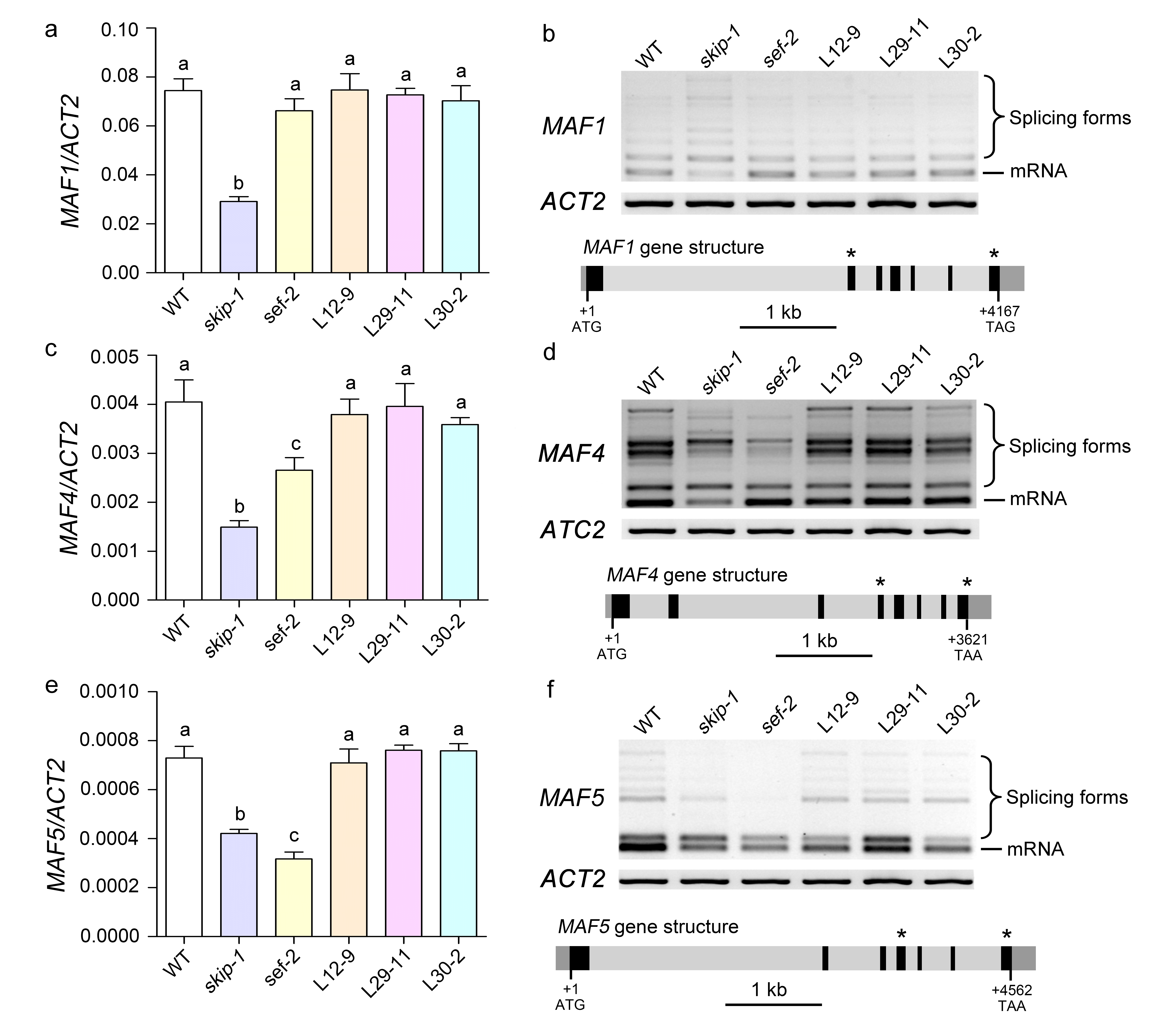

Supplement: Supplementary file 9 — The expression and alternative splicing of MAF1, MAF4, and MAF5 in skip-1 and sef-2 mutants under LD conditions. a, c, e MAF1, MAF4, and MAF5 expression in WT, skip-1, sef-2, and three of the skip-1 complemented transgenic lines, including L12-9, L29-11, and L30-2, described in Fig. 1 under LD conditions. b, d, f Alternative splicing of MAF1, MAF4, and MAF5 in WT, skip-1, sef-2, and three of the skip-1 complemented transgenic lines, including L12-9, L29-11, and L30-2, described in Fig. 1 under LD conditions. ACTIN 2 (ACT2) was used as an endogenous control. Three technical replicates were performed. The values are the mean ± s.d. One-way analysis of variance (ANOVA; Tukey’s multiple comparison test) was performed for data in a, c, and e. Statistically significant differences are indicated by different lowercase letters (P < 0.05). There are statistically significant differences between all non-identical letters. (TIF 8262 kb) [file 12915_2017_422_MOESM9_ESM.tif]

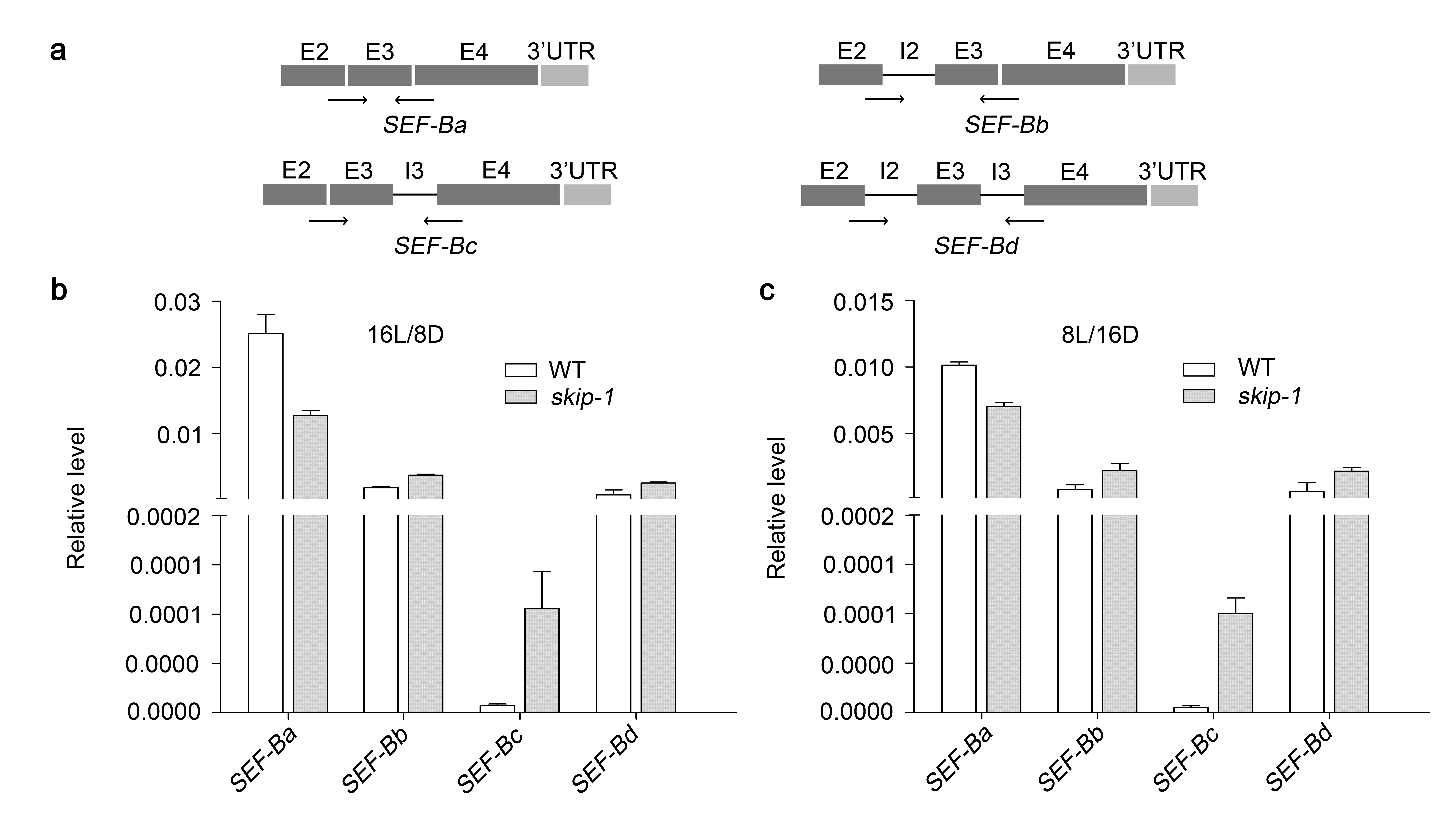

Supplement: Supplementary file 10 — SKIP, a splicing factor, is essential for the splicing of SEF pre-mRNA. a Locations of the primer pairs used to amplify mature SEF mRNA and its alternatively spliced isoforms. SEF-Ba, SEF-Bb, SEF-Bc, and SEF-Bd represent the mature mRNA, isoform containing the second intron, isoform containing the third intron, and isoform containing the second and third introns of the SEF-B fragment. The black arrows indicate the location of primers. b and c The levels of mature SEF mRNA and alternatively spliced isoforms in WT and skip-1 plants under LD and SD conditions as determined by qRT-PCR. Three technical replicates were performed. The values are the mean ± s.d. in b and c. (TIF 1341 kb) [file 12915_2017_422_MOESM10_ESM.tif]

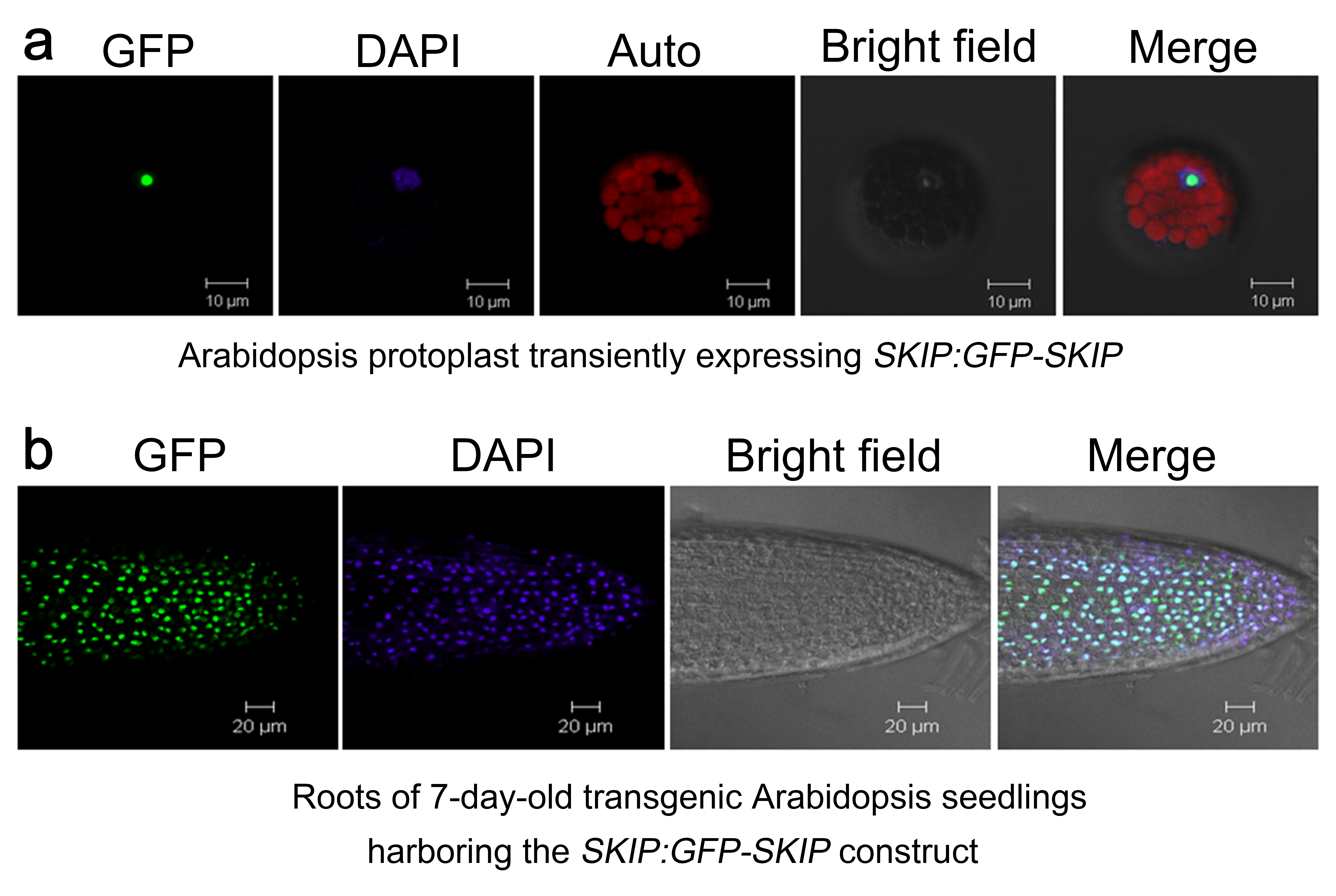

Supplement: Supplementary file 13 — SKIP is localized to the nucleus to perform its function. a A SKIP construct encoding GFP driven by the native SKIP promoter (SKIP:GFP-SKIP) was transiently expressed in Arabidopsis protoplasts. b Roots of 7-day-old seedlings stably transformed with SKIP:GFP-SKIP. The images were produced by laser scanning confocal microscopy. DAPI 4',6-diamidino-2-phenylindole. Scale bar, 10 μm in a and 20 μm in b. (TIF 4964 kb) [file 12915_2017_422_MOESM13_ESM.tif]

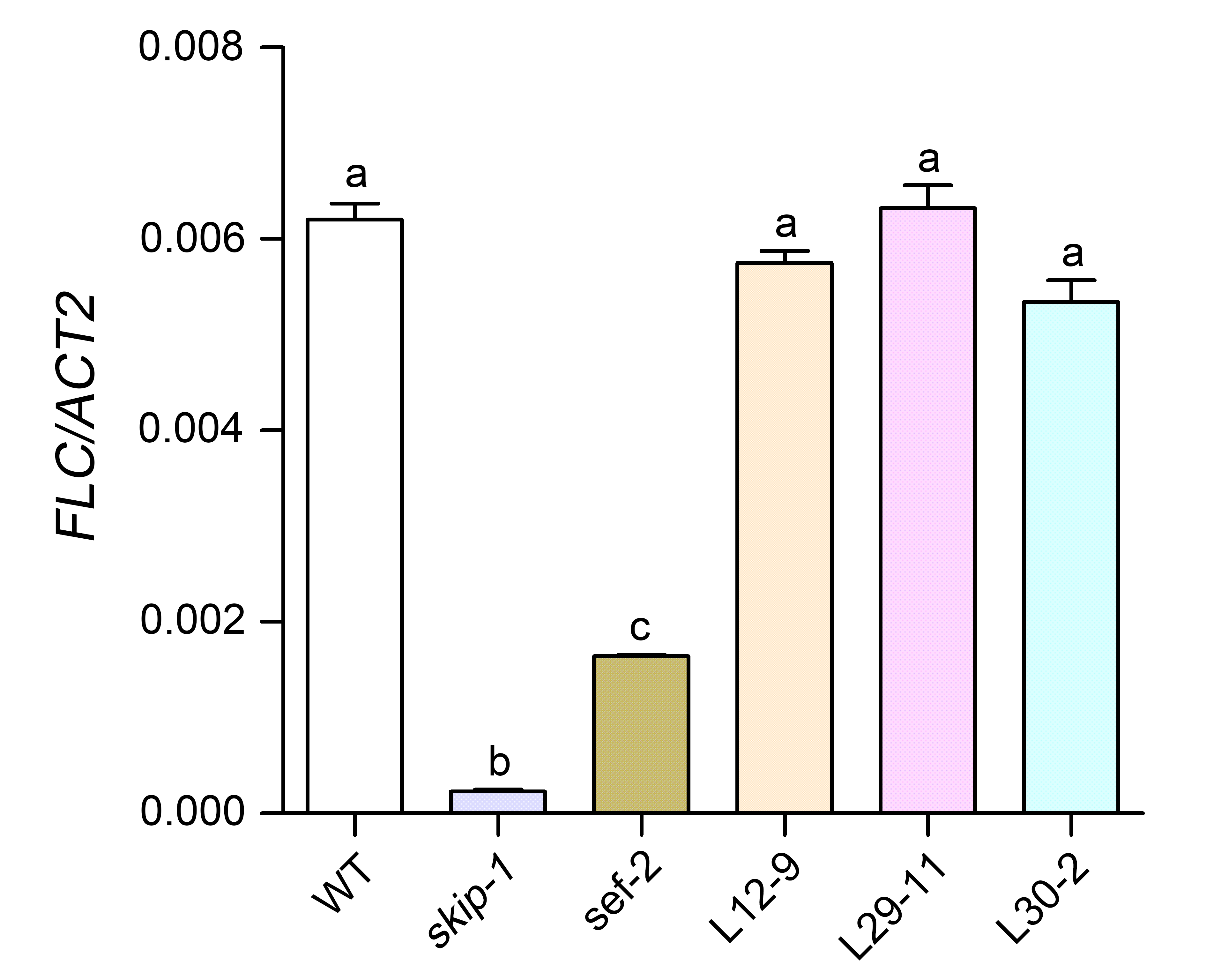

Supplement: Supplementary file 14 — The expression of FLC in WT, skip-1, sef-2, and three of the skip-1 complemented transgenic lines, including L12-9, L29-11, and L30-2, described in Fig. 1 under LD conditions. ACTIN 2 (ACT2) was used as an endogenous control. Three technical replicates were performed. The values are the mean ± s.d. One-way analysis of variance (ANOVA; Tukey’s multiple comparison test) was performed. Statistically significant differences are indicated by different lowercase letters (P < 0.05). There are statistically significant differences between all non-identical letters. (TIF 503 kb) [file 12915_2017_422_MOESM14_ESM.tif]
