# Supplementary material for: SKIP controls flowering time via the alternative splicing of SEF pre-mRNA in Arabidopsis
Source: BMC Biol. 2017 Sep 11;15:80. doi: 10.1186/s12915-017-0422-2 (PMC5594616; doi:10.1186/s12915-017-0422-2)
Supplement: Supplementary file 5 — skip-1 is able to recover the late flowering phenotypes of FRI and fve, and flk mutants under LD conditions. (DOC 43 kb) [file 12915_2017_422_MOESM5_ESM.doc]

**Additional file 5: Table S4.** *skip-1* is able to recover the late flowering phenotypes of *FRI* and *fve*, and *flk* mutants under LD conditions

| Genotype | Rosette leaf number | Cauline leaf number | Days to flower bud emerging (day) | Days to first flower blooming (day) | n |
| --- | --- | --- | --- | --- | --- |
| WT | 11.20 ± 0.70^1^ | 2.20 ± 0.55 | 21.50 ± 1.00 | 28.20 ± 0.89 | 15 |
| *skip-1* | 6.87 ± 0.63 | 3.60 ± 0.63 | 18.67 ± 1.11 | 27.27 ± 1.49 | 15 |
| *FRI*(Col-0) | 85.67 ± 4.55 | ------ | ------ | ------ | 15 |
| *skip-1/FRI*^2^ | 23.31 ± 3.55 | 8.35 ± 2.59 | 37.88 ± 2.7 | 45.88 ± 3.6 | 26 |
| WT | 10.94 ± 0.85 | 2.44 ± 0.51 | 23.81 ± 1.28 | 30.00 ± 1.03 | 16 |
| *skip-1* | 6.21 ± 0.43 | 3.86 ± 0.36 | 18.57 ± 1.02 | 28.14 ± 1.70 | 14 |
| *fve* | 22.00 ± 1.04 | 3.50 ± 0.52 | 33.42 ± 0.79 | 40.67 ± 1.78 | 12 |
| *skip-1/fve^2^* | 8.24 ± 0.54 | 2.86 ± 0.57 | 23.10 ± 1.30 | 31.52 ± 1.12 | 21 |
| WT | 11.00 ± 1.70 | 2.68 ± 0.78 | 22.95 ± 2.06 | 29.68 ± 2.15 | 22 |
| *skip-1* | 7.68 ± 0.57 | 2.73 ± 0.46 | 21.09 ± 1.15 | 28.55 ± 1.37 | 22 |
| *flk* | 21.11 ± 2.05 | 4.28 ± 0.75 | 32.06 ± 2.21 | 39.00 ± 2.66 | 27 |
| *skip-1/flk^2^* | 13.07 ± 1.30 | 3.52 ± 1.05 | 27.44 ± 1.65 | 35.89 ± 2.24 | 18 |

1. The data are mean ± s.d.. 2. *skip-1/FRI*, *skip-1/fve*, and *skip-1/flk* are the double mutants between *skip-1* and *FRI*, *fve*, *flk* mutants.
