# Supplementary material for: SKIP controls flowering time via the alternative splicing of SEF pre-mRNA in Arabidopsis
Source: BMC Biol. 2017 Sep 11;15:80. doi: 10.1186/s12915-017-0422-2 (PMC5594616; doi:10.1186/s12915-017-0422-2)
Supplement: Supplementary file 6 — Overexpression of FLC is able to recover the early flowering phenotypes of skip-1 under LD conditions. (DOC 34 kb) [file 12915_2017_422_MOESM6_ESM.doc]

**Additional file 6: Table S5.** Overexpression of *FLC* is able to recover the early flowering phenotypes of *skip-1* under LD conditions

| Genotype | Rosette leaf number | Cauline leaf number | Days to flower bud emerging (day) | Days to first flower blooming (day) | n |
| --- | --- | --- | --- | --- | --- |
| WT | 15.32 ± 1.76^1^ | 3.90 ± 0.80 | 29.10 ± 0.99 | 37.01 ± 1.63 | 18 |
| *skip-1* | 8.65 ± 0.87 | 3.45 ± 0.65 | 23.89 ± 0.77 | 35.21 ± 1.67 | 20 |
| D1-12^2^ | 14.75 ± 1.23 | 5.26 ± 0.85 | 30.45 ± 1.01 | 42.56 ± 1.35 | 18 |
| D4-9 | 15.23 ± 1.67 | 3.78 ± 0.96 | 29.98 ± 0.89 | 40.65 ± 1.89 | 21 |
| D16-23 | 13.89 ± 0.89 | 4.50 ± 0.69 | 29.23 ± 1.23 | 42.79 ± 1.13 | 23 |

1. The data are mean ± s.d.. 2. D1-12, D4-9, and D16-23 are the *skip-1* transgenic lines harboring *p35S*:*FLC* construct.
