# Supplementary material for: SKIP controls flowering time via the alternative splicing of SEF pre-mRNA in Arabidopsis
Source: BMC Biol. 2017 Sep 11;15:80. doi: 10.1186/s12915-017-0422-2 (PMC5594616; doi:10.1186/s12915-017-0422-2)
Supplement: Supplementary file 7 — FLC expression as measured by qRT-PCR in the skip-1 transgenic lines under LD conditions. (DOC 32 kb) [file 12915_2017_422_MOESM7_ESM.doc]

**Additional file 7: Table S6. *FLC* expression as measured by qRT-PCR^1^ in the *skip-1* transgenic lines under LD conditions**

| Genotype | WT | *skip-1* | D1-12^2^ | D4-9 | D16-23 |
| --- | --- | --- | --- | --- | --- |
| Rep. 1^3^ | 0.001447 | 0.000619 | 8.321817 | 9.329967 | 7.985302 |
| Rep. 2 | 0.001399 | 0.000680 | 7.964588 | 9.476183 | 7.873227 |
| Rep. 3 | 0.001262 | 0.000666 | 7.575661 | 8.619357 | 7.785041 |
| Mean | 0.001369 | 0.000655 | 7.954022 | 9.141836 | 7.881190 |
| s.d. | 0.000096 | 0.000032 | 0.373190 | 0.458348 | 0.100368 |

1. *ACT2* was used as an endogenous control. 2. D1-12, D4-9, and D16-23 are the *skip-1* transgenic lines harboring *p35S*:*FLC* construct. 3. Rep. 1, Rep. 2, and Rep. 3 represent three technical replicates respectively.
