# Supplementary material for: SKIP controls flowering time via the alternative splicing of SEF pre-mRNA in Arabidopsis
Source: BMC Biol. 2017 Sep 11;15:80. doi: 10.1186/s12915-017-0422-2 (PMC5594616; doi:10.1186/s12915-017-0422-2)
Supplement: Supplementary file 11 — SEF regulates flowering time through alternative splicing under LD conditions. (DOC 38 kb) [file 12915_2017_422_MOESM11_ESM.doc]

**Additional file 11: Table S7.** SEF regulates flowering time through alternative splicing under LD conditions

| Genotype | | Rosette leaf  number | Cauline leaf number | Day to flower bud emerging (day) | Day to first flower blooming (day) | n |
| --- | --- | --- | --- | --- | --- | --- |
| WT | | 9.08 ± 0.58^1^ | 1.89 ± 0.32 | 23.92 ± 1.13 | 30.12 ± 0.81 | 41 |
| *sef-2* | | 6.00 ± 0.67 | 2.38 ± 0.49 | 19.36 ± 0.80 | 25.73 ± 0.95 | 44 |
| *sef-2*/  *35S:SEFc* | C5-15^2^ | 7.17 ± 0.38 | 2.20 ± 0.41 | 21.68 ± 0.80 | 27.73 ± 0.78 | 30 |
|  | C13-5 | 7.32 ± 0.53 | 2.47 ± 0.50 | 22.46 ± 0.51 | 27.94 ± 0.83 | 36 |
|  | C22-8 | 7.72 ± 0.66 | 2.25 ± 0.44 | 21.90 ± 0.81 | 27.57±0.77 | 37 |
| *sef-2*/  *35S:wtSEF_IR_* | W2-2^3^ | 8.35 ± 0.84 | 2.09 ± 0.28 | 22.53 ± 0.51 | 28.85 ± 0.80 | 33 |
|  | W23-7 | 8.23 ± 0.43 | 2.24 ± 0.43 | 21.79 ± 0.74 | 27.78 ± 0.90 | 36 |
|  | W33-2 | 8.23 ± 0.42 | 2.43 ± 0.50 | 22.04 ± 0.94 | 28.15 ± 0.79 | 40 |
| *sef-2*/  *35S:mSEF_IR_* | M2-13^4^ | 5.87 ± 0.55 | 2.45 ± 0.50 | 19.67 ± 0.71 | 26.09±0.50 | 40 |
|  | M33-13 | 5.47 ± 0.51 | 2.25 ± 0.44 | 19.56 ± 0.79 | 26.63 ± 0.76 | 30 |
|  | M39-8 | 5.29 ± 0.47 | 2.00 ± 0.47 | 19.85 ± 1.04 | 26.78 ± 1.09 | 20 |

1. The data are mean ± s.d.. 2. C5-15, C13-5, and C22-8 are the *sef-2* transgenic lines harboring *p35S:SEFc* construct. 3. W2-2, W23-7, and W33-2 are the *sef-2* transgenic lines harboring *p35S:wtSEF_IR_* construct. 4. M2-13, M33-13, and M39-8 are the *sef-2* transgenic lines harboring *p35S:wtSEF_IR_* construct.
