# Supplementary material for: SKIP controls flowering time via the alternative splicing of SEF pre-mRNA in Arabidopsis
Source: BMC Biol. 2017 Sep 11;15:80. doi: 10.1186/s12915-017-0422-2 (PMC5594616; doi:10.1186/s12915-017-0422-2)
Supplement: Supplementary file 12 — SEF regulates flowering time through alternative splicing under SD conditions. (DOC 40 kb) [file 12915_2017_422_MOESM12_ESM.doc]

**Additional file 12: Table S8.** SEF regulates flowering time through alternative splicing under SD conditions

| Genotype | | Rosette leaf  number | Cauline leaf number | Day to flower bud emerging (day) | Day to first flower blooming (day) | n |
| --- | --- | --- | --- | --- | --- | --- |
| WT | | 55.43 ± 4.07^1^ | 9.43 ± 1.09 | 92.86 ± 6.80 | 109.14 ± 5.87 | 14 |
| *sef-2* | | 34.36 ± 2.41 | 8.67 ± 1.15 | 67.86 ± 5.92 | 82.90 ± 4.58 | 14 |
| *sef-2*/  *35S:SEFc* | C5-15^2^ | 38.92 ± 4.27 | 9.63 ± 0.74 | 64.54 ± 4.82 | 80.00 ± 5.90 | 14 |
|  | C13-5 | 34.41 ± 3.24 | 9.78 ± 1.30 | 70.88 ± 6.87 | 82.00 ± 5.75 | 14 |
|  | C22-8 | 38.88 ± 2.68 | 10.20 ± 1.15 | 66.50 ± 4.32 | 78.82 ± 5.85 | 15 |
| *sef-2*/  *35S:wtSEF_IR_* | W2-2^3^ | 45.18 ± 4.64 | 9.65 ± 1.32 | 78.69 ± 5.13 | 92.94 ± 6.68 | 16 |
|  | W23-7 | 45.06 ± 4.53 | 11.25 ± 1.44 | 79.07 ± 5.56 | 92.40 ± 6.60 | 15 |
|  | W33-2 | 42.80 ± 2.93 | 11.44 ± 1.59 | 75.86 ± 4.07 | 86.56 ± 4.19 | 14 |
| *sef-2*/  *35S:mSEF_IR_* | M2-13^4^ | 35.23 ± 4.02 | 10.36 ± 1.12 | 69.67 ± 5.52 | 81.25 ± 7.90 | 12 |
|  | M33-13 | 34.57 ± 4.55 | 8.83 ± 1.40 | 72.38 ± 4.35 | 85.30 ± 4.00 | 10 |
|  | M39-8 | 35.08 ± 3.50 | 8.55 ± 0.69 | 69.27 ± 4.91 | 83.08 ± 3.90 | 13 |

1. The data are mean ± s.d.. 2. C5-15, C13-5, and C22-8 are the *sef-2* transgenic lines harboring *p35S:SEFc* construct. 3. W2-2, W23-7, and W33-2 are the *sef-2* transgenic lines harboring *p35S:wtSEF_IR_* construct. 4. M2-13, M33-13, and M39-8 are the *sef-2* transgenic lines harboring *p35S:wtSEF_IR_* construct.
