# Supplementary material for: SKIP controls flowering time via the alternative splicing of SEF pre-mRNA in Arabidopsis
Source: BMC Biol. 2017 Sep 11;15:80. doi: 10.1186/s12915-017-0422-2 (PMC5594616; doi:10.1186/s12915-017-0422-2)
Supplement: Supplementary file 15 — Primers used in ChIP assays. (DOC 43 kb) [file 12915_2017_422_MOESM15_ESM.doc]

**Additional file 15: Table S9.** Primers used in ChIP assays

| Name | Forward 5’-3’ | Reverse 5’-3’ |
| --- | --- | --- |
| *FLC* B1 | GCATTAGGTTGTTCCCTCCAAAC | GCCCTACCCATGACTAACGTGAG |
| *FLC* B2 | CGGAGTGGGTGAAACTGATTACTG | CATCAAAACTTCTTGGCACAGCTC |
| *FLC* B3 | GTTCGGGAGATTAACACAAATAATAAAGG | GAAAACAAGCTGATACAAGCATTTCAC |
| *FLC* B4 | TGGGGGTAAACGAGAGTGATG | GCAATAGTTCAATCCGTATCGTAGG |
| *FLC* B5 | TGTTCTCAATTCGCTTGATTTCTAGT | GCCCGACGAAGAAAAAGTAGATAG |
| *FLC* B6 | CGAGCACGCATCAGATCG | GGCGGATCTCTTGTTGTTTCTC |
| *FLC* B7 | GACGTGCATATACAAATCCAAGAGAAC | CTTTGAATCACAATCGTCGTGTG |
| *FLC* B8 | GCTGGACCTAACTAGGGGTGAAC | CCTCTTTGGTACGGATCTATAATGAATC |
| *FLC* B9 | CTTGAGGACAAGGTTTTTTCCAG | GGCTTCCTCATACTTATGGTTATCTG |
| *FLC* B10 | CCTCTACTGTCCAGATTGTTTCTATGC | TGTCATCACATTGTGGCTCATC |
| *FLC* B11 | CATCTCTCCAGCCTGGTCAAG | GGGCTATGAAAATTGCGGTATG |
| *FLC* B12 | CCTCTCCGTGACTAGAGCCAAG | CTTCAACATGAGTTCGGTCTGC |
| *FLC* B13 | CCGGTTGTTGGACATAACTAGG | CTCTACCAAACCCAGACTTAACCAG |
| *FLC* B14 | CCTTGGATAGAAGACAAAAAGAGAAAGTG | AGGTGACATCTCCATCTCAGCTTC |
| *FLC* B15 | ACAAAGTTCATCAACCTTTTGTCTT | GCGGTTGAAATCAAAATCCA |
| *MAF4* B1 | TACAGGTGGGGAAGAAGACG | AAAGCTCAGCCGTTGATGAT |
| *MAF4* B2 | CCGGTTATCTTTCAGGGTCA | TCCAACAAATCGATTCGACA |
| *MAF4* B3 | TGTTGCTTAAGGAAAAGGGTTC | AAATCCGAAAATGTGCGTGT |
| *MAF4* B4 | TCCATCACAAAAGATGGGTTT | CCTTGCTCAAGGCTTGTTTT |
| *MAF4* B5 | TTCCCCCTTTTCCTCTGATT | AGCTGCTCTTCCAGGGACTT |
| *MAF4* B6 | TCGTGGCAAAGTTAAGACCA | TTTTTGTGCAACCTGACAGC |
| *MAF5* B1 | CTCAGCCCAATGTTTTGGTT | AACTCAAGGACGTTGATTGGA |
| *MAF5* B2 | CGAGTGCCATTTGTTTTTGA | TCCAAGAACTACTTTTGACCATCG |
| *MAF5* B3 | TCCAATTTGGCTTATTCTGTGA | TGAAATTCATGTTTGCAAGTGA |
| *MAF5* B4 | AGATTTCGGATCTTGCCTGA | GGCCGGGATAGTGGATAAAA |
| *MAF5* B5 | GACAGGATCAGAAGTTTCGAATG | TTGGCCTCAAGGTTCTTCAC |
