# Supplementary material for: Correction to: SKIP controls flowering time via the alternative splicing of SEF pre-mRNA in Arabidopsis
Source: BMC Biol. 2019 Mar 20;17:25. doi: 10.1186/s12915-019-0640-x (PMC6425691; doi:10.1186/s12915-019-0640-x)
Supplement: Supplementary file 1 — Table S10. Primers used in the studies (DOCX 18 kb) [file 12915_2019_640_MOESM1_ESM.docx]

Additional file 16: Table S10. Primers used in the studies

| Primer name | Sequence | Note |
| --- | --- | --- |
| ActinF | GGTGTCATGGTTGGTATGGGTC | ACT2 qRT-PCR |
| ActinR | CCTCTGTGAGTAGAACTGGGTGC |  |
| FLCF | CCTCTCCGTGACTAGAGCCAAG | FLC qRT-PCR |
| FLCR | AGGTGACATCTCCATCTCAGCTTC |  |
| MAF1F | TCACCTTAAACTCAAAGCCTGATTC | MAF1 qRT-PCR |
| MAF1R | CAAACTCTGATCTTGTCTCCGAAG |  |
| MAF2F | GAGAGAGAAACGAAGAAAAAAAAAGAAGC | MAF2 qRT-PCR |
| MAF2R | GATGAGACCATTGCGTCGTTTG |  |
| MAF3F | TATCTTCCTCGCGCCAATG | MAF3 qRT-PCR |
| MAF3R | AGCACAAGAACTCTGATATTTGTCTAC |  |
| MAF4F | GCTTCTCAAGTAACCACCATCAC | MAF4 qRT-PCR |
| MAF4R | CTTGGATGACTTTTCCGTAGCAG |  |
| MAF5F | GCTGATTTTTCATCATCCTGATTC | MAF5 qRT-PCR |
| MAF5R | GAAACTTCTGATCCTGTCTTCCAAG |  |
| COF | CATTAACCATAACGCATACATTTCA | CO qRT-PCR |
| COR | CTCCTCGGCTTCGATTTCTC |  |
| FTF | TAGTAAGCAGAGTTGTTGGAGACG | FT qRT-PCR |
| FTR | GGGAAGGCCGAGATTGTAGAT |  |
| TSFF | GGAGATTGGAGGAGACGACTTCAG | TSF qRT-PCR |
| TSFR | GCACACCACCTCATTGCCAAAG |  |
| SOC1F | TCATCTTCTTCCTTCTCTTTCTTTCTTC | SOC1 qRT-PCR |
| SOC1R | CGAGGATAAAGGGTTTGTGTATGTATAG |  |
| FDF | CGCGCTAGGAAACAGGCTTAT | FD qRT-PCR |
| FDR | GCTGCAGCCATTTTTAACTGATC |  |
| FCAF | cttctgagctagctcagctcttg | FCA q/semi-RT-PCR |
| FCAR | CAGATCCAGCCCACTGTTGTTTAC |  |
| FYF | caccatcagctaccaccatcatc | FY q/semi-RT-PCR |
| FYR | AGGATTCATGCCACCTTGCATTC |  |
| FLKF | caaccacctcctagacatgatag | FLK q/semi-RT-PCR |
| FLKR | GTAACTTGCTGAGCGACCATTG |  |
| FLDF | gatatggtgctgtgtacagttc | FLD q/semi-RT-PCR |
| FLDR | CATACGGAAAGAGCATTGCAAC |  |
| FPAF | cctaataatgcagcgcctcaag | FPA q/semi-RT-PCR |
| FPAR | TGCTTCTCCATTGTACAGTCCATTG |  |
| FVEF | ctcgtctggccttcactctcttg | FVE q/semi-RT-PCR |
| FVER | CTGCAGCAACCCTTGGCTTAAC |  |
|  |  |  |
| LDF | tgattcgcagagtcaattgcttc | LD q/semi-RT-PCR |
| LDR | GAATCATCTGCAGAAGTAAGCAAC |  |
| VRN1F | CCGTTACTCCATTCGCATTGGTTAT | VRN1 q/semi-RT-PCR |
| VRN1R | TCGGCATCTTCATCTTCAAGGTCTT |  |
| VRN2F | CTTTCAACCTTCGCTCTCTAGG | VRN2 q/semi-RT-PCR |
| VRN2R | ACCACATAGCATAGAGCAAAATGG |  |
| VIN3F | GACTTTCTTCGGGTGTGCAT | VIN3 q/semi-RT-PCR |
| VIN3R | CAAAACAACCTGAAACCTGTGA |  |
| FLCD1F | ATGGGAAGAAAAAAACTAGAAATCAAG | FLC D1 semi-RT-PCR |
| FLCD1R | CTTGGCTCTAGTCACGGAGAGG |  |
| FLCD2F | CACTATGAGCTACTTGAACTTGTGGATAG | FLC D2 semi-RT-PCR |
| FLCD2R | AGGTGACATCTCCATCTCAGCTT |  |
| FLCFBamHI | GGATCCATGGGAAGAAAAAAACTAGAAATCAA | 35S:FLC construct |
| FLCRSacI | GAGCTCCTAATTAAGTAGTGGGAGAGTCACCG |  |
| SEF-AaF | GACAATCGAACTCAGGCTGCTA | SEF-Aa qRT-PCR |
| SEF-AaR | GCTTCTTCTGTAAGTAACCGAGATCAT |  |
| SEF-AbF | ATCGAACTCAGGTGAAATTGTTGA | SEF-Ab qRT-PCR |
| SEF-AaR | GCTTCTTCTGTAAGTAACCGAGATCAT |  |
| SEF-AaF | GACAATCGAACTCAGGCTGCTA | SEF-Ac qRT-PCR |
| SEF-AcR | TCTTCTGTAAGTAACCTACCAACTCAGAA |  |
| SEF-BaF | GAAGATGATGATCTCGGTTACTTACAGA | SEF-Ba qRT-PCR |
| SEF-BaR | AGACTCCAAATTTGCCTCTTGC |  |
| SEF-BbF | AGATGATGATCTCGGTGAATAATCA | SEF-Bb qRT-PCR |
| SEF-BaR | AGACTCCAAATTTGCCTCTTGC |  |
| SEF-BbF | AGATGATGATCTCGGTGAATAATCA | SEF-Bc qRT-PCR |
| SEF-BcR | AAAGACTCCAAATTTGCCTAAAACA |  |
| SEFAF | ATGGAGGAAGAGATGTCGAACC | SEF-A semi-RT-PCR |
| SEFAR | AGCTCAAGGAAGGATTTAGGAGC |  |
| SEFBF | ATGATGAAGAAGCTTCTCTTGATGA | SEF-B semi-RT-PCR |
| SEFBR | ATAGCGACGAGAAGACGAACTC |  |
| SEFAF | ATGGAGGAAGAGATGTCGAACC | SEF semi-RT-PCR |
| SEFBR | ATAGCGACGAGAAGACGAACTC |  |
| SEFcF | TCTAGAATGGAGGAAGAGATGTCGAACC | 35S:SEFc construct and 35S:wtSEF_IR_ construct |
| SEFcR | GGATCCCTATGCAACAAATTTCTGACAACG |  |
| SEFcF | TCTAGAATGGAGGAAGAGATGTCGAACC | 35S:muSEF_IR_ construct |
| SEFcL5R | TCAACAATTTCGTCAGAGTTCGAT |  |
| SEFcL5F | CAACGACAATCGAACTCTGACGAAATTG |  |
| SEFcL3R | TCTAGCCTAGCTATAGCAGTGAAGAATCAACC |  |
| SEFcL3F | GGTTGATTCTTCACTGCTATAGCTAGG |  |
| SEFcR | GGATCCCTATGCAACAAATTTCTGACAACG |  |
| SEF-AbF | ATCGAACTCAGGTGAAATTGTTGA | RNA IP |
| SEF-AaR | GCTTCTTCTGTAAGTAACCGAGATCAT |  |
